# Osteoarthritis pain inversely correlates with histidine and glutamine following CSF and serum profiling

**DOI:** 10.1186/s13075-026-03839-1

**Published:** 2026-06-19

**Authors:** Isobel K. Dunstan, Line J. H. Rasmussen, Fay Probert, Dorte A. Olsen, Jonna S. Madsen, Jesper Eugen-Olsen, Thomas Peter Enggaard, Claus Varnum, Kate L. Lambertsen, Daniel C. Anthony, Morten Rune Blichfeldt-Eckhardt

**Affiliations:** 1 Department of Pharmacology, Medical Sciences Division, University of Oxford, Mansfield Road, Oxford, OX1 3QT UK; 2 Department of Chemistry, Mathematical, Physical & Life Sciences Division, University of Oxford, Oxford, UK; 3 Department of Clinical Research, Copenhagen University Hospital, Hvidovre, Denmark; 4 Department of Biochemistry and Immunology, Lillebaelt Hospital, University Hospital of Southern Denmark, Vejle, Denmark; 5 Department of Regional Health Research, Faculty of Health Sciences, University of Southern Denmark, Odense, Denmark; 6 Department of Cardiology, Copenhagen University Hospital-Herlev and Gentofte Hospital, Herlev, Denmark; 7 Department of Anaesthesia, Multidiciplinary Pain Center, Copenhagen University Hospital, Copenhagen, Denmark; 8 Department of Orthopedic Surgery, Lillebaelt Hospital, University Hospital of Southern Denmark, Vejle, Denmark; 9 Department of Neurology, Odense University Hospital, Odense, Denmark; 10 Department of Neurobiology Research, Institute of Molecular Medicine, University of Southern Denmark, Odense, Denmark; 11 BRIDGE, Brain Research-Inter Disciplinary Guided Excellence, Department of Clinical Research, University of Southern Denmark, Odense, Denmark; 12 Department of Anaesthesia, Lillebaelt Hospital, University Hospital of Southern Denmark, Vejle, Denmark

**Keywords:** Metabolomics, Osteoarthritis, Neuroinflammation, Chronic pain biomarkers, 1H NMR spectroscopy, Cytokines, Cerebrospinal fluid, C-reactive protein

## Abstract

**Background:**

Osteoarthritis is a leading cause of pain and disability, yet the biological processes linking peripheral joint pathology with central pain mechanisms and wider symptom burden remain poorly defined.

**Methods:**

We performed an integrated metabolomic and inflammatory analysis of cerebrospinal fluid and serum obtained from patients with osteoarthritis (*n* = 81) and healthy pain-free controls (*n* = 70). Proton nuclear magnetic resonance spectroscopy was used for metabolomic profiling, alongside targeted protein assays for inflammatory mediators. Orthogonal partial least squares discriminant analysis was applied to assess separation between groups and to determine diagnostic accuracy. Associations between metabolites and clinical outcomes, including pain intensity, disability and sleep disturbance, were examined, with adjustment for age and BMI.

**Results:**

Clear separation between osteoarthritis and healthy pain-free control participants was observed in both biofluids, with classification accuracies of 87% for serum and 89% for cerebrospinal fluid. Reduced serum histidine, glutamine, albumin (lysyl) and lysine distinguished osteoarthritis from healthy controls. In cerebrospinal fluid, osteoarthritis was characterised by higher lactate and glutamate and lower glucose and glutamine compared to controls. Combining metabolomic data with inflammatory proteins increased diagnostic accuracy to 90% and remained significant after matching for age and BMI. Reductions in serum histidine and glutamine were consistent across subgroups, including stratification by pain severity. These metabolites correlated inversely with pain intensity, disability, sleep disturbance and overall symptom impact, and were more markedly altered in women, who also reported greater symptom burden.

**Conclusions:**

Osteoarthritis is associated with a distinct pattern of peripheral and central metabolic disturbance. Histidine and glutamine emerge as promising biomarkers related to pain and clinical severity, highlighting metabolic pathways as potential targets for improved stratification and intervention in osteoarthritis pain.

**Supplementary Information:**

The online version contains supplementary material available at https://doi.org/10.1186/s13075-026-03839-1.

## Background

Osteoarthritis (OA) is a complex joint disease characterised by cartilage degradation, synovial inflammation, and subchondral bone changes [1–3]. It is the most prevalent joint disorder in older adults and a major cause of disability [2, 4]. OA pathogenesis involves multiple factors, including mechanical influences, aging, and genetic predisposition [1]. Synovial inflammation and subchondral bone alterations are increasingly recognised as key contributors to OA onset and progression [5, 6]. These changes can precede visible cartilage degeneration and are associated with pain and functional impairment [6]. The subchondral bone undergoes uncoupled remodelling, leading to osteosclerosis in advanced stages [5]. Current treatments aim to relieve symptoms and improve joint function, but no curative therapy exists [7]. Emerging strategies targeting cytokine inhibition and signalling pathways offer potential avenues for disease modification [8], but personalised approaches to prevention and treatment, tailoring interventions to distinct OA phenotypes are likely to be necessary [9].

Sex differences are a recognised feature of OA, with women experiencing higher prevalence, pain intensity, and poorer clinical outcomes compared to men. Women account for 60% of OA cases globally [10] and report more severe pain, functional limitations, and poorer surgical outcomes [10, 11]. These differences are influenced by hormonal factors, joint anatomy, central pain processing, and psychosocial aspects [12, 13]. The interplay between inflammatory markers and pain also varies by sex, suggesting distinct biological mechanisms underlying OA progression [12]. Addressing these disparities is crucial for tailoring interventions and improving treatment outcomes across sexes [14, 15]. More broadly, structural joint damage correlates only modestly with pain severity, with many individuals exhibiting a marked discordance between radiographic burden and symptom intensity. This highlights that mechanisms beyond local joint pathology, including those influencing pain perception, contribute to the clinical phenotype [16].

Consistent with this dissociation between structural damage and symptom burden, there is increasing evidence that OA is accompanied by systemic alterations detectable beyond the joint, including changes in circulating inflammatory mediators, metabolic proteins, and regulatory molecules such as microRNAs in serum [17]. These findings suggest that OA reflects broader biological processes linked to pain and disease expression, rather than a purely local joint disorder. In this context, the potential of cerebrospinal fluid (CSF) biomarkers is an emerging area of interest, particularly given the central role of pain processing in OA [18, 19]. Indeed, studies have identified elevated levels of inflammatory proteins in CSF of OA patients, indicating neuroinflammation and supporting a central component to OA pain [20, 21]. Interestingly, some CSF biomarkers, particularly those with neuroprotective effects, are associated with reduced pain intensity and milder symptoms [21]. Specific proteins like monocyte chemoattractant protein 1 (MCP1) and interleukin (IL)-8 have been found elevated in CSF, suggesting their role in neuroimmune signalling [20]. However, the biochemical composition of CSF in OA remains largely unexplored. This likely reflects the practical challenges of CSF collection, particularly in healthy control samples, making paired central and peripheral analyses in human cohorts scarce.

Metabolomics, the comprehensive measurement of small molecule metabolites within a biofluid, has emerged as a powerful approach for defining disease phenotypes and their underlying biological processes. In OA, this approach has revealed alterations in amino acid, lipid, and energy metabolism, with nuclear magnetic resonance (NMR) studies of biofluids, particularly synovial fluid, showing promise for biomarker discovery [22–24]. Here, we sought to test the hypothesis that OA is accompanied by a measurable metabolic disturbance in both serum and CSF, and that this disturbance relates to the pain phenotype. To address this, we undertook a multiomics analysis integrating ¹H NMR metabolomics and targeted protein measurements from paired blood and CSF samples. The study had three objectives: first, to compare the metabolic and inflammatory profiles of OA and healthy pain-free controls in serum and CSF; second, within OA, to determine whether the key discriminating metabolites and proteins were associated with clinical measures of pain and disability; and third, to explore whether these relationships differed by sex, given the higher symptomatic burden observed in women.

## Methods

### Participants

Participants aged 18–80 years who were able to understand and respond to the questionnaires were recruited as part of The Danish Pain Research Biobank (DANPAIN Biobank), as previously described [25]. For the current study, samples were included from two groups: individuals with osteoarthritic pain scheduled for hip or knee arthroplasty surgery at a regional hospital (*n* = 81), and pain-free volunteers recruited locally (*n* = 70). The pain-free participants had no acute or chronic pain conditions and were screened to exclude any recent pain symptoms. Detailed recruitment procedures and cohort characteristics have been described previously [25]. A summary of participant characteristics for the present study cohort is provided in Table S1, derived in part from previously published data. The study was conducted in accordance with the Declaration of Helsinki, with written informed consent obtained from all participants, and approval granted by the relevant regional ethics committees, as described previously [25].

### Sample collection

Participants fasted prior to sampling (≥ 2 h in healthy pain-free; ≥6 h for food and ≥ 2 h for liquids in the OA group, consistent with perioperative protocols), and in OA participants, samples were obtained pre-operatively prior to arthroplasty. Blood (24 mL) and CSF (7 mL) samples were obtained following standard protocols. Serum was collected in plain tubes and stored at room temperature for 30 min before centrifugation. All samples were centrifuged at 2,000 G for 10 min at 4 °C, aliquoted on ice blocks, and stored at − 80 °C. CSF was collected via lumbar puncture using atraumatic needles and stored at − 80 °C within 30 min of collection. Separate aliquots were analysed for neuroimmune biomarkers and for metabolomics to avoid freeze-thaw effects. CSF contaminated with blood was excluded, and samples were stored under identical conditions to blood.

### Clinical and pain assessments

Functional and psychological impacts were assessed using validated instruments, including the Symptom Impact Questionnaire (SIQR) [26], Major Depression Inventory (MDI), Generalized Anxiety Disorder (GAD-7), Insomnia Severity Index (ISI), EuroQol visual analogue scale (EQ VAS), and the Oxford Hip or Knee score (OHS/OKS) [27]. Pain intensity and interference were quantified using a Numeric Rating Scale (NRS) [28] and Pain Disability Index (PDI) [29], respectively. All clinical, pain, and questionnaire-based assessments were completed within 24 h of blood and CSF sampling, and in all cases prior to surgery for participants undergoing arthroplasty. Questionnaires were administered electronically using a standardised clinical registry platform, as previously described [25].

### Analysis of neuroimmune biomarkers

A panel of biomarkers in CSF and blood was quantified using multiplex assays from Mesoscale Discovery as described elsewhere [30]. High-sensitivity C-reactive protein (hsCRP, mg/L) was analyzed with the CRP high sensitive ELISA (TECAN, IBL International GmbH, Hamburg, Germany) according to the manufacturer’s instructions. Serum suPAR (suPAR, ng/mL) was analyzed with the suPARnostic^®^ AUTO Flex ELISA (ViroGates A/S, Birkerød, Denmark) according to manufacturer’s instructions. Neurofilament light (NfL) and glial fibrillary acidic protein (GFAP) in CSF and serum were measured blinded to clinical data using a commercially available NfL and GFAP 2-plex assay on the Single Molecule Array (Simoa^®^) HD-X Analyzer (Quanterix, Billerica, MA, USA) [31]. Further details on all assays, including limits of detection and assay ranges are listed in the Supplementary methods S1.1.

### Sample processing and 1H NMR data collection

Serum (150 µL) and CSF (100 µL) samples were mixed with 400 µL and 450 µL of NMR buffer (75 mM sodium phosphate buffer prepared in D_2_O, pH 7.4), respectively. All samples were then placed in a 5 mm NMR tube and measured using a 700-MHz Bruker AVII spectrometer operating at 16.4 T, equipped with a ^1^H (^13^C/^15^N) TCI cryoprobe (Chemistry Research Laboratory, Department of Chemistry, University of Oxford), as described previously [32, 33]. Further details can be found in Supplementary methods S1.2.

### 1H NMR data processing

Topspin 4.1.4 (Bruker) was used to phase, baseline correct, and reference all spectra to the lactate methyl resonance at δ = 1.33 ppm. ACD/Labs Spectrus Processor Academic Edition 12.01 (Advanced Chemistry Development, Inc.) was used to manually bin each resonance signal, excluding all noise from analysis. The integrals of these bins were sum normalised and exported to R version 4.3.0 [34]. Metabolite assignment was conducted through a mix of in-house databases, literature reviews, 2D total correlation spectroscopy (TOCSY) experiments, and spiking reference compounds where needed [35, 36].

### Multivariate statistical analysis

Orthogonal partial least squares discriminant analysis (OPLS-DA) was used to model group differences, as it enables separation of predictive variation related to class discrimination from orthogonal (non-class-related) variation, improving interpretability in high-dimensional metabolomics data. Specifically, the ropls package in R [37] was used to conduct orthogonal partial least squares discriminant analysis (OPLS-DA) between pain-free and OA predictor variables (i.e. all serum and CSF metabolites and proteins from the targeted panels, z-scaled). Model performance was evaluated against a null distribution generated by random permutation of class labels to assess the likelihood of spurious separation. Descriptions of the model building and cross validation scheme can be found in Supplementary methods S1.3.

### Univariate statistical analysis

Univariate analyses such as Student’s T test and two-way ANOVA were carried out in R (v 4.3.0). The significance threshold was set to ∗*p* < 0.05, ∗∗*p* < 0.01, ∗∗∗*p* < 0.001, ∗∗∗∗*p* < 0.0001. All correlations were tested via Pearson correlational analyses, using the Benjamini-Hochberg method to correct for false discovery rate at 0.05. Univariate Receiver Operating Characteristic (ROC) analyses and multivariate ROC analyses on a combination of features using logistic regression were determined using the pROC package in R.

## Results

### Distinct serum and CSF metabolite profiles, and their integration with inflammatory markers, differentiate osteoarthritis from healthy pain-free controls

**Fig. 1:**
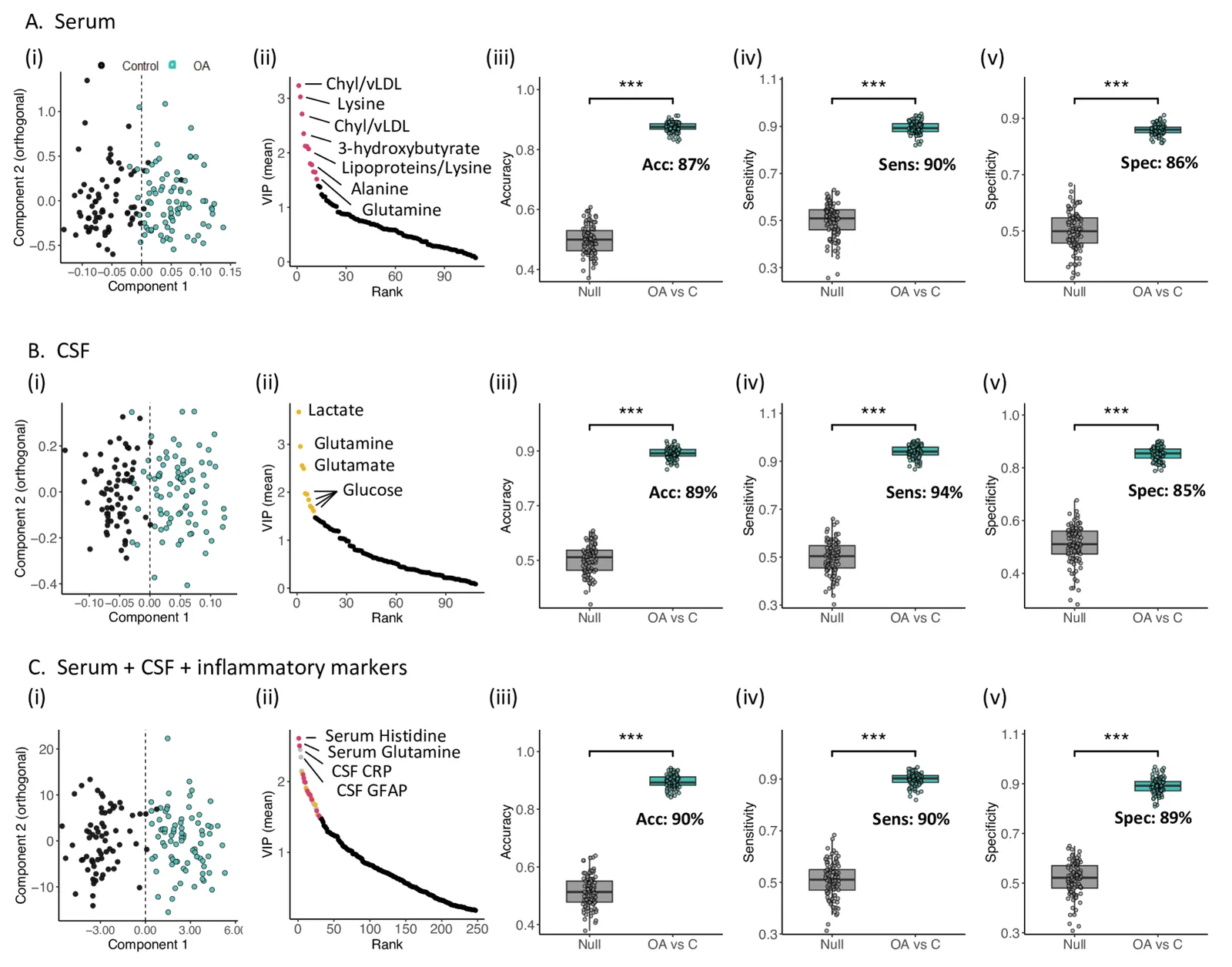
Multivariate models of serum, CSF and combined biofluids distinguish osteoarthritis (OA) from pain-free controls. **A** Serum: (i) OPLS–DA scores plot of serum ¹H NMR data showing separation of OA (turquoise) from healthy pain-free controls (black). (ii) Variable importance in projection (VIP) plot ranking discriminatory serum metabolites, with chylomicron/VLDL, lysine, 3-hydroxybutyrate, alanine and glutamine among the highest contributors. (iii) Accuracy, (iv) sensitivity and (v) specificity of the serum model compared with the permuted null model, showing Acc 87%, Sens 90% and Spec 86% (***p < 0.001). **B** CSF: (i) OPLS–DA scores plot of CSF metabolites showing comparable separation. (ii) VIP plot indicating lactate, glutamine, glutamate and glucose as the main discriminatory CSF variables. (iii–v) Model metrics for CSF, giving Acc 89%, Sens 94% and Spec 85%, all superior to the null model (***p < 0.001). **C** Serum + CSF + inflammatory markers: (i) OPLS–DA scores plot for the integrated dataset. (ii) VIP plot showing serum histidine and serum glutamine, together with CSF CRP and CSF GFAP, as the dominant variables when compartments are combined. (iii–v) Performance of the integrated model, yielding Acc 90%, Sens 90% and Spec 89%, again exceeding the null distribution (***p < 0.001), indicating additive value of combining peripheral, central and inflammatory readouts. All models were built on z-scaled data and evaluated with repeated cross validation. Supplementary Fig. S1 shows a concordant random forest analysis, and Supplementary Fig. S2 presents box plots for the top serum and CSF VIPs from panels **A** and **B**

We first examined whether untargeted ¹H NMR spectra from single biofluids could discriminate individuals with OA from pain-free healthy controls. OPLS-DA models built on serum profiles showed a clear separation between groups (Fig. 1A(i)), with a classification accuracy of 87%, sensitivity of 90% and specificity of 86%, all significantly better than the null permutation model. The principal discriminatory serum signals were higher mobile lipid-related resonances in the chylomicron/VLDL region and higher 3-hydroxybutyrate in OA compared with controls. In contrast, amino acids including lysine, alanine and glutamine were lower in OA (Fig. 1A(ii) and Supplementary Fig. S2A(i-iv) for boxplots). Model performance metrics are shown in Fig. 1A(iii–v). CSF profiles gave a very similar picture, again separating OA from healthy pain-free controls (Fig. 1B(i)) and doing so with slightly higher diagnostic performance (accuracy 89%, sensitivity 94%, specificity 85%). In CSF, lactate and glutamate were higher in OA compared with healthy controls, while glutamine and glucose were lower. This pattern indicates that metabolic alterations extend to the central compartment as well as the periphery (Fig. 1B(ii) and Supplementary Fig. S2B(i-iv) for boxplots). Model performance metrics are shown in Fig. 1B(iii–v). When serum and CSF metabolites were analysed together with inflammatory and neurodegeneration-related proteins, model performance improved further, with the integrated model achieving 90% accuracy, 90% sensitivity and 89% specificity, driven chiefly by serum histidine and serum glutamine together with CSF CRP and CSF GFAP (Fig. 1C(ii)), with model performance shown in Fig. 1C(iii–v). A random forest approach confirmed that OA and healthy pain-free controls could also be separated using an orthogonal modelling strategy (Supplementary Fig. S1), and the distribution of the top serum and CSF VIPs is shown as box plots in Supplementary Fig. S2. These plots illustrate that, in serum, OA was characterised by higher very low density lipoprotein signals (mobile CH₃ in chylomicron/VLDL) and 3-hydroxybutyrate, together with lower lysine, alanine and glutamine. In CSF, lactate and glutamate were increased, while glutamine and glucose were reduced in OA compared with healthy controls. A summary of cohort characteristics is provided in Supplementary Table S1. Given the perioperative fasting requirements in the OA group, we examined whether time since last meal influenced the key discriminating metabolites; no consistent associations were observed (Supplementary Fig. S3), indicating that fasting duration is unlikely to account for the observed group differences. These findings suggest distinct metabolic alterations in both systemic and, more surprisingly, central compartments in OA.

### Age- and BMI-matched analysis confirms a core OA metabolic-inflammatory signature independent of demographic differences

**Fig. 2:**
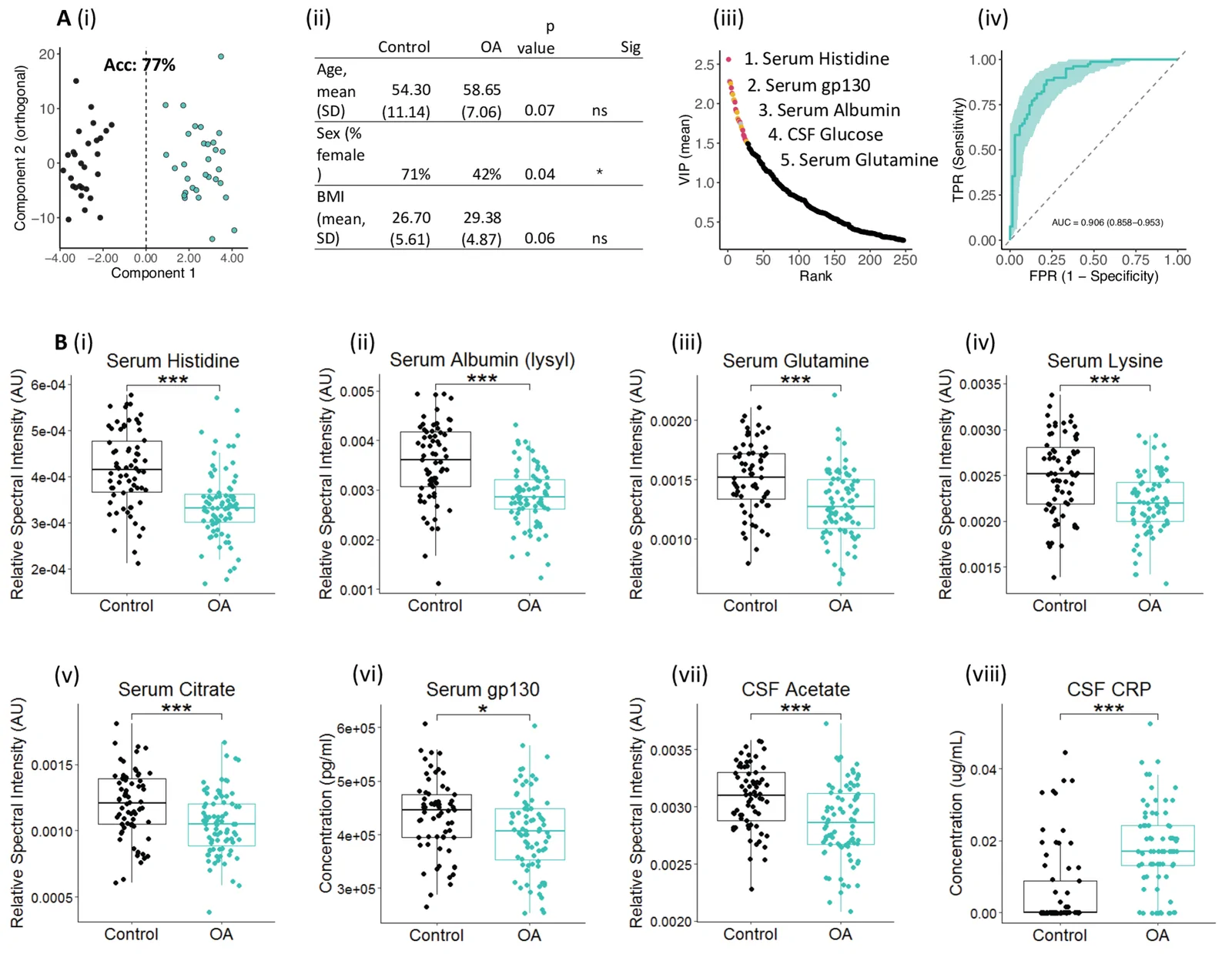
Age and BMI matching confirm that key serum and CSF biomarkers remain altered in osteoarthritis. **A** Matched multivariate model. (i) OPLS–DA scores plot for the age- and BMI-matched subset (OA *n* = 31, controls *n* = 28) showing that OA (turquoise) remains separable from healthy pain-free controls (black), with an accuracy of 77%, indicating that group discrimination is not driven solely by demographic differences. (ii) Characteristics of the matched subset showing no significant differences in age or BMI between groups, with a modest imbalance in sex, which notably was not present in the full cohort. (iii) VIP plot from the matched model identifying serum histidine as the highest-ranking variable, followed by serum gp130, serum albumin (lysyl), CSF glucose and serum glutamine. (iv) ROC curve derived from refitting the matched-surviving variables in the full cohort, yielding an AUC of 0.906 (95% CI 0.858–0.953), which shows that a reduced, demographically robust panel is sufficient to distinguish OA from healthy controls. **B** Distribution of matched-surviving variables in the full cohort. Box plots show lower concentrations of serum histidine (i), serum albumin (lysyl) (ii), serum glutamine (iii), serum lysine (iv) and serum citrate (v) in OA compared with controls, together with higher serum gp130 (vi), higher CSF acetate (vii) and higher CSF CRP (viii) in OA. These variables were selected because they were discriminatory in both the original and the matched models, so are unlikely to be confounded by age or BMI. Asterisks denote statistical significance: *p < 0.05, **p < 0.01, ***p < 0.001

To exclude the possibility that the discrimination observed in the full cohort was driven by age and BMI, we rebuilt the model in an age and BMI matched subset (OA *n* = 31, controls *n* = 28). In this restricted sample, age and BMI no longer differed significantly between groups and only a modest imbalance in sex remained (which was not present in the full cohort, Fig. 2A(ii)), yet the OPLS-DA model still separated OA from healthy pain-free controls with an accuracy of 77% (Fig. 2A(i)), significantly better than the null model (Supplementary Fig. S4). The VIP ranking from this matched model again placed serum histidine at the top, followed by serum gp130, serum albumin (lysyl moiety), CSF glucose and serum glutamine (Fig. 2A(iii)), several of which were also among the highest ranking variables in the full integrated model (Fig. 1C), indicating that these signals are not explained by demographic confounders. Consistent with this, correlation analyses in the full cohort showed only weak associations between these key metabolites and age or BMI (Supplementary Fig. S5), further supporting that the observed metabolic signature is not driven by these demographic factors.

We next compared variables identified in the full and age- and BMI-matched analyses and retained those that were significantly different between OA and healthy pain-free controls in both cohorts based on univariate testing (Supplementary Fig. S4). This approach ensured that only features reproducible across analyses and robust to demographic confounding were carried forward. This reduced panel still achieved a strong diagnostic performance, with an AUC of 0.906 (95% CI 0.858–0.953; Fig. 2A(iv)), showing that a small, demographically robust signature is sufficient to distinguish OA from healthy pain-free individuals. The direction and magnitude of these key variables in the whole cohort are illustrated in Fig. 2B. Serum histidine, albumin (lysyl), glutamine and lysine were lower in OA compared with healthy controls. In contrast, serum citrate, serum gp130, CSF acetate and CSF CRP were higher in OA. Corresponding box plots for the matched subset are provided in Supplementary Fig. S6 and a side by side comparison of VIP scores in the full and matched models is shown in Supplementary Fig. S4B.

### Core metabolic signature associates with pain burden, symptom impact and sleep disturbance

**Fig. 3:**
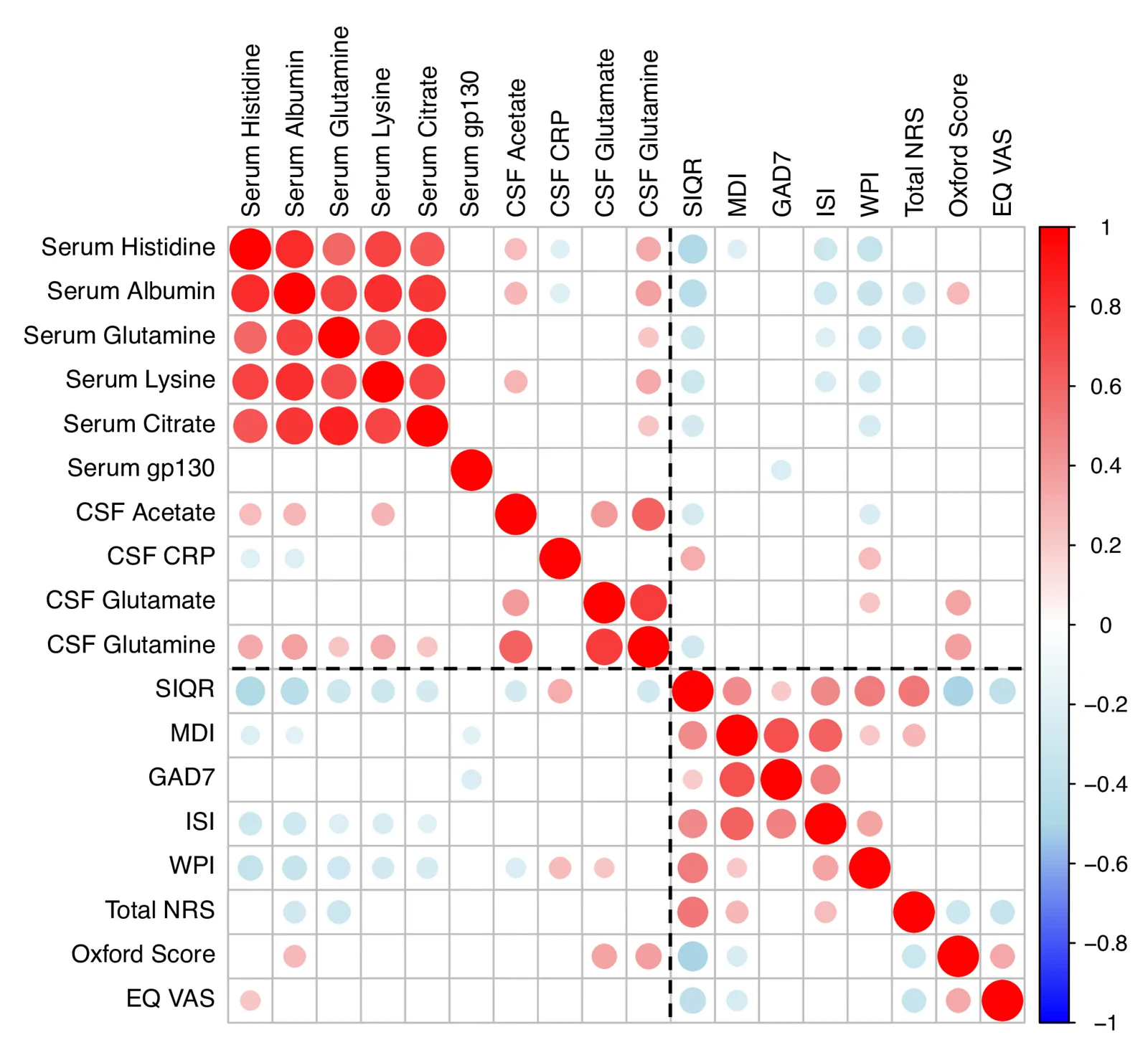
Correlations between top discriminating metabolites and clinical outcomes in osteoarthritis. Pearson correlation coefficients are shown for the serum and CSF variables that remained discriminatory after age/BMI matching (serum histidine, serum albumin (lysyl), serum glutamine, serum lysine, serum citrate, serum gp130, CSF acetate, CSF CRP, CSF glutamate and CSF glutamine) and for clinical and symptom scores. Circle colour and size indicate the direction and strength of the correlation. Correlations shown above the diagonal remain significant after false discovery rate correction. The horizontal and vertical dashed lines separate biochemical variables from clinical variables. Lower serum histidine, albumin and glutamine were associated with higher symptom burden, including SIQR, ISI and total NRS, while higher CSF CRP, CSF acetate and CSF glutamate tracked with worse clinical scores. Clinical measures: SIQR, Symptom Impact Questionnaire Revised; MDI, Major Depression Inventory; GAD7, Generalised Anxiety Disorder 7 item scale; ISI, Insomnia Severity Index; WPI, Widespread Pain Index; Total NRS, composite numeric rating scale totalled from average pain score, pain during exercise, pain at rest, and strongest pain; Oxford Score, Oxford hip/knee score; EQ VAS, EuroQol visual analogue scale

To determine whether the demographically robust metabolic and inflammatory variables were clinically meaningful, we examined their correlations with pain and disability measures within the whole cohort. The restricted panel of serum metabolites (histidine, albumin lysyl resonance, glutamine, lysine and citrate) was strongly intercorrelated, forming a coherent serum metabolic module, and this module correlated inversely with several symptom scores, including the SIQR, total NRS, ISI and WPI (Fig. 3; *p* values in Table S3). In other words, lower serum histidine, albumin and glutamine were associated with greater symptom impact, higher pain ratings and poorer sleep. In contrast, central and inflammatory markers that survived matching, in particular CSF CRP, CSF acetate and CSF glutamate, showed positive correlations with these same clinical measures, consistent with a contribution of central immune activation to symptom severity. Anxiety and mood scores (GAD7, MDI) followed the same pattern, although the associations were weaker. A broader correlation matrix including the full inflammatory panel reproduced these relationships and showed additional links to CSF TNFRI/II and GFAP (Supplementary Fig. S8), supporting the view that OA pain is accompanied by a coupled metabolic inflammatory disturbance across serum and CSF.

### Serum metabolites track intra-disease variation in pain intensity

**Fig. 4:**
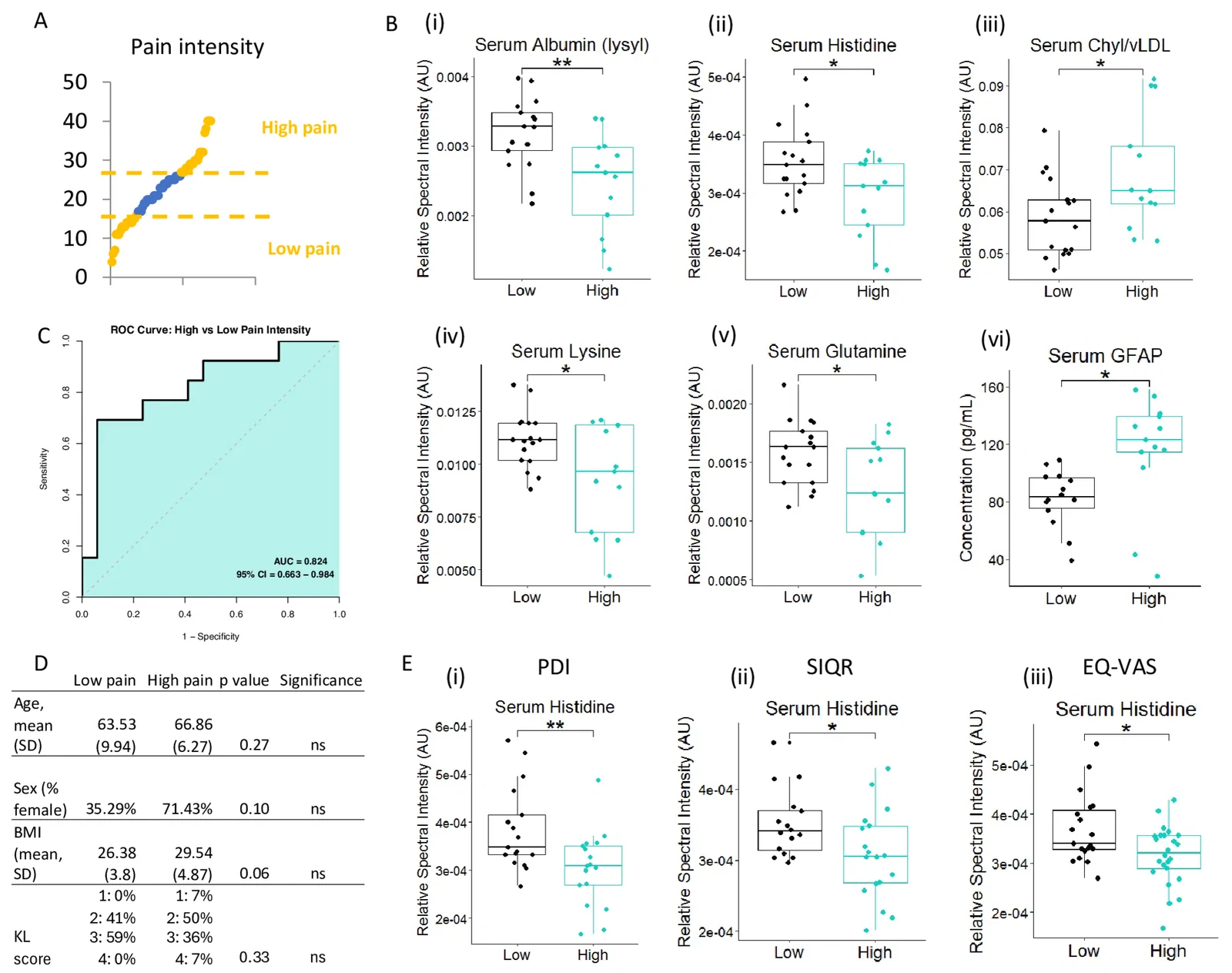
Serum metabolites distinguish between high and low pain intensity within osteoarthritis. **A** Definition of pain groups. OA participants were stratified into low and high pain according to the lower and upper quartiles of the total Numeric Rating Scale (NRS), with higher values indicating greater pain intensity. **B** Metabolites associated with pain. Box plots show that serum albumin (lysyl) (i), serum histidine (ii), and serum lysine (iv) and serum glutamine (v) were significantly lower in the high pain group, whereas the chylomicron/VLDL signal (iii) and serum GFAP (vi) were higher. **C** Discriminative performance. A logistic model built from the pain sensitive metabolites in panel **B** separated high from low pain OA with an AUC of 0.824 (95% CI 0.663–0.984). **D** Group characteristics. Age, sex distribution, BMI and Kellgren-Lawrence (KL) grade did not differ significantly between high and low pain groups, indicating that the metabolic differences are related to pain state rather than structural severity. **E** Clinical relevance. Serum histidine remained significantly lower in participants with higher Pain Disability Index (PDI) (i) and SIQR scores (ii), and in those with lower EQ-VAS ratings (iii), confirming that this metabolite tracks broader symptom impact. Continuous variables were tested with unpaired t tests, categorical variables with Fisher’s exact test or the Monte Carlo extension for KL grade. Asterisks denote significance: *p < 0.05, **p < 0.01

We next asked whether the metabolic signature identified above also captured symptom severity within OA itself. OA participants were stratified into low and high pain groups using the lower and upper quartiles of the total NRS (Fig. 4A). Several of the key serum variables showed clear, directional differences between these groups (Fig. 4B(i–vi)). Serum albumin (lysyl resonance), histidine, lysine and glutamine were all lower in the high pain group compared to the low pain group. In contrast, both the chylomicron/VLDL signal and serum GFAP, likely derived from chondrocytes [38], were increased in the high pain group. A logistic model built only from these pain-sensitive variables discriminated high from low pain OA with an AUC of 0.824 (95% CI 0.663 to 0.984; Fig. 4C), suggesting that a small serum panel may have utility for phenotyping painful OA. Importantly, age, sex distribution, BMI and Kellgren-Lawrence scores did not differ between the high and low pain groups (Fig. 4D), indicating that the identified metabolic signature reflects the pain phenotype rather than structural disease severity. This observation is consistent with the well-described discordance between radiographic burden and symptom intensity in OA. Consistent with this, serum histidine also associated with broader measures of impact, showing lower values in participants with higher Pain Disability Index and SIQR scores and with poorer EQ-VAS ratings (Fig. 4E). Together, these data show that the metabolic disturbance identified at disease level is graded by pain intensity and is therefore clinically meaningful.

### Female osteoarthritis shows a heavier symptom burden and a more pronounced metabolic-inflammatory disturbance

**Fig. 5:**
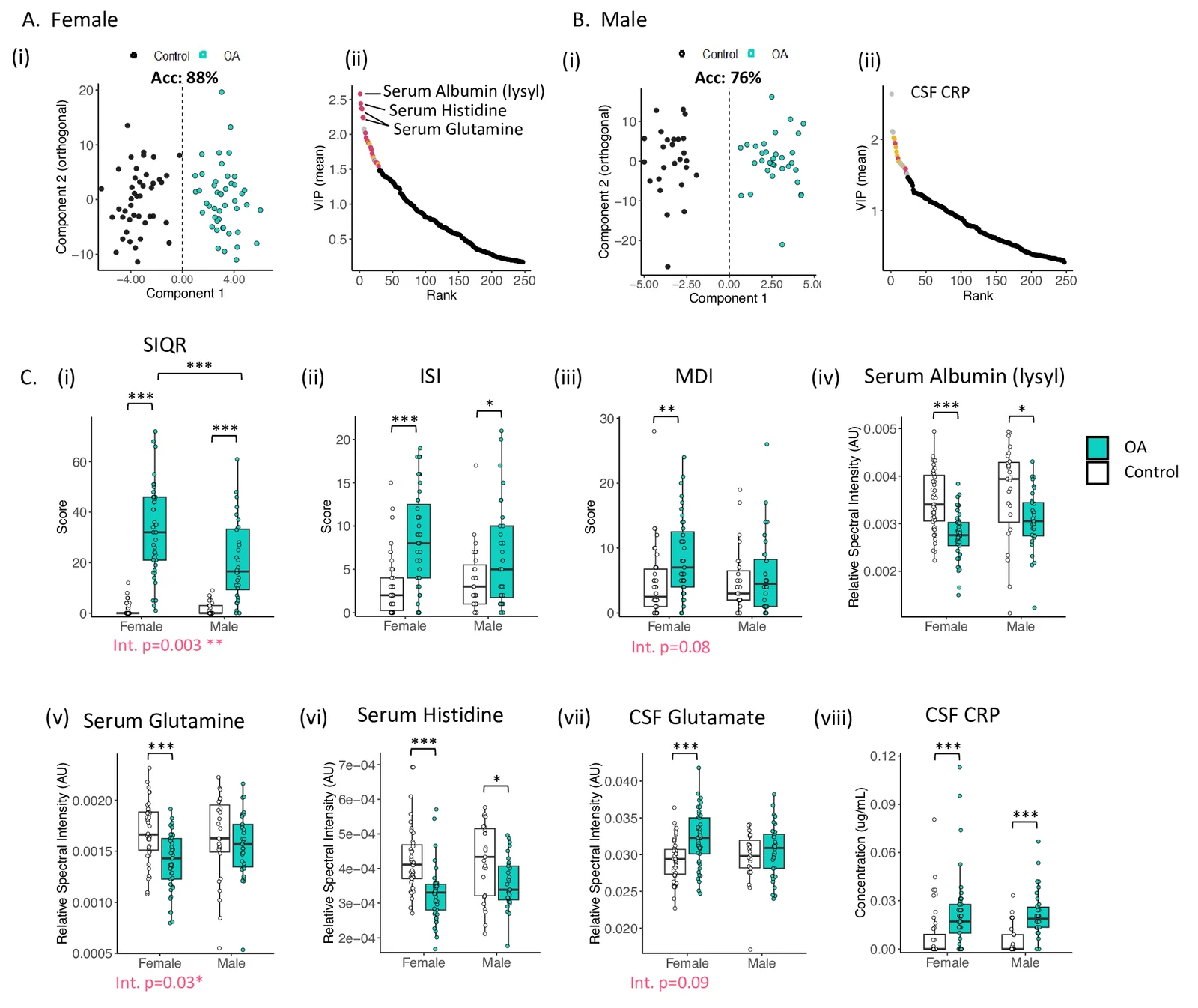
Sex-specific differences in clinical and biofluid profiles in osteoarthritis. **A** Female participants. (i) OPLS-DA scores plot of the integrated serum, CSF, and inflammatory dataset showing clear separation of osteoarthritis (OA, turquoise) from healthy controls (black), with an accuracy of 88%. (ii) VIP plot identifying serum albumin (lysyl), serum histidine, and serum glutamine as the highest-ranking discriminatory variables in females. **B** Male participants. (i) OPLS-DA scores plot also separated OA from healthy controls, although with lower accuracy (76%). (ii) VIP plot showing CSF CRP as the top-ranking variable in males. **C** Clinical outcomes by sex and diagnosis. Compared with sex-matched controls, women with OA reported higher Symptom Impact Questionnaire Revised (SIQR) scores (i), higher Insomnia Severity Index (ISI) scores (ii), and higher Major Depression Inventory (MDI) scores (iii). Two-way ANOVA showed a significant diagnosis × sex interaction for SIQR and a trend for ISI (interaction *p* values shown in pink). **C**, iv–viii) Biofluid variables by sex and diagnosis. Women with OA showed larger reductions in serum albumin (lysyl) (iv), serum glutamine (v), and serum histidine (vi) than men. CSF glutamate was increased in females with OA only (vii), while CSF CRP was elevated in both men and women with OA (viii). Data are shown as box plots with overlaid points. Asterisks denote significance following two-way ANOVA with post hoc testing (*p < 0.05, **p < 0.01, ***p < 0.001). Full two-way ANOVA results are provided in Supplementary Table S2

Because women are disproportionately affected by symptomatic OA, we examined whether the discriminatory signature differed by sex [21]. Two-way ANOVA demonstrated a significant effect of diagnosis on both age (*p* < 0.001) and BMI (*p* = 0.004), with OA participants being older and having higher BMI than healthy controls, while no significant effects of sex or diagnosis-by-sex interactions were observed (Table S2). When analysed separately, OPLS-DA models built from female participants showed robust separation of OA from healthy pain-free controls (accuracy 88%), whereas the model in males, although still significant, was weaker (accuracy 76%) (Fig. 5A, B; Supplementary Fig. S7). The top VIPs in women were the same amino acid related serum variables that survived age/BMI matching, in particular serum albumin (lysyl), serum histidine and serum glutamine, whereas in men CSF CRP rose to the top of the ranking (Fig. 5A(ii), 5B(ii)). Clinically, women with OA reported higher SIQR, ISI and, to a lesser extent, MDI scores than men, and for SIQR and ISI there were significant diagnosis by sex interactions, indicating that the disease effect is larger in females (Fig. 5C(i–iii); Supplementary Table S2). Consistent with this, the metabolite changes that defined OA in the whole cohort, notably lower serum glutamine, histidine and albumin (lysyl), were clearly expressed in women but were weaker or absent in men (Fig. 5C(iv–vi)). Interestingly, CSF glutamate was elevated in females with OA only, potentially related to their higher pain and symptom scores (Fig. 5C(vii)). While CSF CRP was the top driver of the male OPLS-DA model, two-way ANOVA revealed it was significantly increased in both men and women with OA (Fig. 5C(viii)). Taken together, these findings suggest that in women the OA phenotype is driven more by metabolic and excitatory changes that track symptom burden and men in this cohort display a similar, but less pronounced phenotype.

## Discussion

This study asked whether OA has a reproducible biofluid signature, whether that signature relates to pain, and whether it differs by sex. Using untargeted ¹H NMR in serum and CSF, supported by targeted inflammatory markers, we showed that OA can be distinguished from healthy pain-free controls with high accuracy, and that this signal persists after age and BMI matching. The same small set of variables, notably lower serum histidine, also tracked clinical severity, since OA cases with the greatest pain and symptom impact had the most divergent metabolite values. Finally, these effects were most pronounced in women, indicating that combined serum-CSF profiling can capture sex-specific pathways contributing to symptomatic OA. Importantly, these findings reinforce the view of OA as a condition characterised by systemic metabolic disturbance, rather than solely a localised joint disease.

### Metabolic alterations in OA

Our results reveal distinct metabolic signatures in OA, characterised by increases in VLDL and 3-hydroxybutyrate and decreases in lysine, alanine, histidine, and glutamine in serum, along with elevated lactate and glutamate and reduced glutamine and glucose in CSF. These results are consistent with growing evidence suggesting that metabolic dysregulation, particularly in amino acid and lipid metabolism, is linked to inflammation and joint degradation in OA [39]. Chondrocyte metabolism is known to be affected by environmental stressors, which can lead to shifts between metabolic pathways and mitochondrial dysfunction [40]. Furthermore, obesity and metabolic syndrome, often comorbid with OA, exacerbate these metabolic changes through complex interactions of biomechanical, inflammatory, and metabolic factors [41]. These findings support the emerging concept of OA as a metabolic disease, with lipids and adipokines playing crucial roles in cartilage degradation [42, 43]. Similarly, reductions in amino acids such as lysine and glutamine may indicate their increased utilisation in pro-inflammatory and metabolic pathways [43].

The differentiation between serum and CSF metabolite profiles provides further support that OA involves both systemic and central metabolic alterations. Elevated lactate and glutamate in CSF are consistent with altered energy metabolism and excitotoxicity, processes linked to central sensitisation and neuroinflammation. While glutamate-related pathways have been implicated in synovial fluid and fibroblast metabolism [44–46], this study extends these observations to the CNS. Exposure of rat spinal cord slices to CSF from OA patients has previously been shown to affect neuronal excitability, potentially altering nociceptive processing at the spinal level [47]. Elucidating the altered composition of OA CSF here suggests that glutamate signalling may contribute to peripheral nociceptive transduction and inflammation in OA [48]. Additionally, reactive astrocytes and their release of lactate have been implicated in chronic pain development and central sensitisation [49–51]. Notably, the relationship between serum and CSF signals was not uniform. Certain pathways, particularly glutamate and glutamine metabolism, appeared to be shared across compartments and were associated with both central and peripheral inflammatory markers, suggesting a common metabolic-immune axis. In contrast, many CSF-derived variables contributing to discrimination in the full model were not retained after age and BMI matching, whereas a smaller subset of metabolites, including serum histidine and glutamine alongside CSF glucose and acetate, remained robust. This pattern indicates that while a broader CSF signal is detectable, only a subset of central metabolic changes are tightly linked to disease status independent of demographic influences. Taken together, these findings support a model in which systemic metabolic disturbance interacts with, but is not identical to, central processes involved in pain, consistent with central-peripheral crosstalk rather than simple reflection of one compartment by the other.

### Diagnostic potential of biomarker integration

Combining serum and CSF metabolites with a targeted protein panel improved the diagnostic accuracy of OA models, reaching 90%. This integration highlights the value of a multi-modal biomarker approach in capturing the complex and multifactorial nature of OA. Previous research has shown the limitations of single biomarkers in diagnosing OA due to the heterogeneity of the disease [52]. By incorporating markers of inflammation, neurodegeneration, and joint degradation, our model achieved a higher discriminatory power and highlighted unique relationships between metabolites and proteins in CSF and serum. The persistence of certain metabolites, such as reduced serum histidine and glutamine, in both the full and matched cohorts suggests their robustness as potential biomarkers of OA. Histidine is an essential amino acid with anti-inflammatory properties [53]. Indeed, we show here that serum histidine levels are inversely correlated with cytokines both in the serum and CSF (Fig. S8). Others have also highlighted histidine as a potential metabolomic biomarker for knee OA in which the ratio of branched-chain amino acids to histidine seems to be important [54].

### Pain-associated metabolic changes

One of the key findings of this study is the association of specific serum metabolites with OA pain intensity. Decreases in serum histidine, glutamine, lysine, and albumin (lysyl moiety) were observed in participants with high pain intensity, alongside an increase in serum GFAP.

The observed reduction in histidine and glutamine in high-pain groups may reflect their roles in modulating oxidative stress and inflammatory responses. Histidine has been shown to scavenge reactive oxygen species (ROS) and inhibit inflammatory pathways in various studies [53, 55]. It can reduce fluid accumulation and protect intestinal tissue during inflammation [55], as well as inhibit IL-8 secretion in intestinal epithelial cells [56]. Glutamine serves as a substrate for glutathione synthesis, which is crucial for antioxidant activities and immune cell function [57, 58]. Both amino acids have demonstrated potential in modulating respiratory burst in neutrophils [59]. Their immunomodulatory effects make them promising candidates for therapeutic interventions in various conditions, including inflammation, infection, and oxidative stress-related disorders [53, 60]. The elevation of serum GFAP, potentially derived from chondrocytes, suggests a link between joint damage and systemic markers of neurodegeneration, further supporting the interconnectedness of peripheral and central mechanisms in OA pain [61]. Importantly, these metabolic differences were observed in the absence of differences in structural burden between pain-stratified groups, indicating that they relate to symptom severity rather than radiographic disease and are consistent with the recognised discordance between joint pathology and pain in OA. The correlation between these metabolic changes and clinical pain scores, such as the NRS and SIQR, provides a compelling case for the use of serum metabolites as objective measures of pain severity. This could address the current reliance on subjective pain assessments in clinical practice and trials, improving the precision of pain management strategies.

### Sex differences in OA

Female participants exhibited more severe OA symptoms and greater metabolic changes compared to men, consistent with broader findings of higher pain prevalence, worse functional outcomes, and distinct inflammatory profiles [10]. The greater reductions in serum histidine and glutamine, as well as the notably higher CSF glutamate observed in females may reflect sex-specific differences in inflammatory and metabolic processes. Oestrogen influences cartilage metabolism, immune responses, and pain perception, particularly postmenopause, exacerbating OA pathology [62]. Systematic reviews suggest oestrogen replacement therapy may lower OA prevalence by modulating cartilage turnover [63, 64]. Furthermore, metabolic imbalances influenced by oestrogen contribute to OA progression [65]. Psychosocial factors, including anxiety and depression – affecting ~ 20% of OA patients [66] - correlate moderately with pain severity and are more common in women [67]. While the causal link remains unclear, evidence suggests interventions such as cognitive-behavioural approaches may alleviate OA pain and disability [68, 69]. The pronounced metabolite changes in women highlight the need to consider sex differences in OA research and treatment. Tailored interventions that address the unique metabolic and inflammatory pathways in females could improve clinical outcomes and reduce the sex disparity in OA burden. However, we did not have information on menopausal status and therefore, cannot determine the extent to which these effects are driven by post-menopausal hormonal changes.

### Implications for clinical practice and research

This study provides a foundation for the development of biomarker-based diagnostic and therapeutic strategies in OA. Multi-modal biomarker panels integrating serum and CSF metabolites with targeted proteins could improve diagnostic accuracy, stratify patients by disease severity, and inform personalised OA management. The identification of robust biomarkers, such as histidine and glutamine, highlights potential therapeutic targets that warrant further investigation. Interventions aimed at restoring these metabolic pathways could mitigate inflammation and pain, offering a new avenue for disease-modifying treatments. Additionally, the sex-specific differences observed in this study call for the inclusion of sex-stratified analyses in future OA research to ensure equitable and effective treatment development. In addition, these biomarkers may have longitudinal utility for tracking disease progression or predicting symptom trajectories, although this will require validation in prospective studies.

### Limitations and future directions

While this study advances our understanding of OA biomarkers, it has several limitations. The cross sectional design precludes causal inference, and the sample size, particularly in the matched analyses, may limit generalisability. In particular, several CSF metabolites that contributed to discrimination in the full cohort were not retained in the age- and BMI-matched analysis, and may reflect reduced statistical power in the smaller matched subset. As a result, additional central metabolic signals may have been missed, and the relative contribution of CSF-derived features may be underestimated in the matched models. Furthermore, we were not able to perform an independent external validation, largely because major population resources such as UK Biobank do not include CSF, which makes replication of a paired serum-CSF analysis challenging. At the same time, this also underscores the distinctiveness of the present cohort. Longitudinal studies are needed to define the temporal relationship between these metabolic alterations and OA progression and to test whether the serum variables that track pain can predict future symptom flares. Experimental work to probe the functional roles of histidine, glutamine and the CSF inflammatory markers in OA pathophysiology would also be highly valuable. Finally, we did not have data on menopausal status, which limits interpretation of the observed sex differences given the known influence of oestrogen on inflammation, metabolism, and pain processing.

## Conclusions

In summary, this study identifies distinct metabolic and inflammatory profiles in OA. Specifically, reduced serum histidine and glutamine emerged as key discriminators, correlating inversely with pain intensity and disability scores and should be further explored for their role in OA pathophysiology and pain modulation as well as possible targets for personalised treatment strategies.

## Supplementary Information


Supplementary Material 1.


## Data Availability

The datasets generated and analysed during the current study are not publicly available as they are subject to ethical and governance restrictions associated with the DANPAIN Biobank, but are available from the corresponding author on reasonable request.

## Abbreviations

AUC: Area under the curve
BMI: Body mass index
CRP: C reactive protein
CSF: Cerebrospinal fluid
DANPAIN: Danish Pain Research Biobank
D2O: Deuterium oxide
EQ VAS: EuroQol visual analogue scale
GAD 7: Generalised Anxiety Disorder 7 item scale
GFAP: Glial fibrillary acidic protein
hsCRP: High sensitivity C reactive protein
IL: Interleukin
ISI: Insomnia Severity Index
KL: Kellgren to Lawrence grade
MCP 1: Monocyte chemoattractant protein 1
MDI: Major Depression Inventory
NfL: Neurofilament light chain
NMR: Nuclear magnetic resonance
NRS: Numeric rating scale
OA: Osteoarthritis
OHS: Oxford Hip Score
OKS: Oxford Knee Score
OPLS DA: Orthogonal partial least squares discriminant analysis
PDI: Pain Disability Index
ROC: Receiver operating characteristic
SIQR: Symptom Impact Questionnaire Revised
Simoa: Single Molecule Array
suPAR: Soluble urokinase plasminogen activator receptor
TNF: Tumour necrosis factor
TNFR I: Tumour necrosis factor receptor 1
TNFR II: Tumour necrosis factor receptor 2
TOCSY: Total correlation spectroscopy
VLDL: Very low density lipoprotein
