# Supplementary material for: Osteoarthritis pain inversely correlates with histidine and glutamine following CSF and serum profiling
Source: Arthritis Res Ther. 2026 Jun 19;28:161. doi: 10.1186/s13075-026-03839-1 (PMC13531973; doi:10.1186/s13075-026-03839-1)
Supplement: Supplementary file 1 [file 13075_2026_3839_MOESM1_ESM.pdf]

Supplementary methods

S1.1. Analysis of neuroimmune biomarkers

The panel of biomarkers consisted of tumor necrosis factor (TNF), lymphotoxin-alpha (LTα), IL-8, IL-6, IL-1α, IL-1β, and IL-1 receptor antagonist (IL-1Ra) in CSF and plasma and IL-6 receptor (IL-6R), glycoprotein (gp130), TNF receptor 1 (TNFR1), and TNFR2 in CSF and serum. The CRP assay range was 0-10 mg/L, and the limit of detection was 0.02 mg/L. The CVs reported by the manufacturer were 4.1–6.9% intra-assay and 5.8–6.3% inter-assay.
The detection limit of the suPAR assay was 0.4 µg/L. The CV reported by the manufacturer was 2.3–6.0%. The assay range was 1-15 µg/L. Lower limit of detection for NfL and GFAP were 0.065 ng/L and 0.475 ng/L, respectively. The functional lower limit of quantification were 0.8 ng/L for NfL and 16.6 ng/L for GFAP using serum and 8 ng/L for NfL and 166 ng/L for GFAP using CSF. To evaluate and monitor assay performance over time four quality controls two provided by the manufacturer, one in-house prepared serum pool and one in-house prepared CSF pool were included in each run. The total analytical variation for the included controls were; 9-16 % for NfL and 9-18% for GFAP.

S1.2. Sample Processing and 1H NMR data collection

Serum spectra were acquired using a spin-echo sequence (Carr-Purcell-Meiboom- Gill [CPMG]), 32 data collections, an acquisition time of 1.5 s, a relaxation delay of 2 s, and a fixed receiver gain. CSF spectra were acquired with a 1D ^{1}H with Nuclear Overhauser Effect Spectroscopy (NOESY) presaturation scheme (2 s pre-saturation) for attenuation of the water resonance (34). All samples were run within 9 hours of being thawed.

S1.3. Multivariate Statistical Analysis

Models were validated through external cross-validation, whereby the data was separated into train (90%) and test sets (10%), 10 times for every 100 external fold, producing 1000 models in total. The training data was used to learn discriminatory patterns in the predictor

variables, whereas the test set was used for assessing model prediction parameters (accuracy, sensitivity, and specificity). Significant separation between OA and pain-free samples was determined by comparing to a null distribution, where classes were shuffled randomly to achieve the same sized dataset for direct comparison. The key variables for distinguishing between classes were identified using Variable Importance in Projection (VIP) scores and were investigated using univariate analysis as described below.

	Control (n=70)	Osteoarthritis (n=81)	P value
**Age (years), mean ± SD †**	37.4 ± 16.2	67.1 ± 8.9	<0.001
**Female (%) ‡**	43 (61.4)	46 (56.8)	0.56
**BMI (kg/m²), mean ± SD †**	25.7 ± 4.6	28.4 ± 5.7	0.002
**Smoking status, n (%) ‡**			0.70
No	50 (71.4)	56 (69.1)	
Yes	8 (11.4)	13 (16.0)	
Previous smoking	11 (15.7)	11 (13.6)	
**Alcohol consumption (units/week), n (%) ‡**			0.013
0	30 (42.9)	15 (18.5)	
1-7	32 (45.7)	52 (64.2)	
8-14	5 (7.1)	7 (8.6)	
≥15	2 (2.9)	5 (6.2)	
**Highest education, n (%) ‡**			<0.001
Primary school education	5 (7.1)	19 (23.5)	
Upper secondary education	19 (27.1)	4 (4.9)	
Vocational education/training	6 (8.6)	22 (27.2)	
Short cycle education	5 (7.1)	11 (13.6)	
Vocational bachelor education	24 (34.3)	15 (18.5)	
Master's program	10 (14.3)	3 (3.7)	
Others	1 (1.4)	5 (6.2)	
**Work situation, n (%) ‡**			<0.001
Normal working hours	34 (48.6)	20 (24.7)	
Reduced working hours	0	1 (1.2)	
Sick leave (partly or fulltime)	1 (1.4)	4 (4.9)	
Pension	5 (7.1)	45 (55.6)	
Studying	24 (34.3)	0	
Unemployed	0	2 (2.5)	
Other	6 (8.6)	8 (9.9)	
**Physical activity, n (%) ‡**			<0.001
Primarily sedentary pursuits	10 (14.3)	16 (19.8)	
Physical activity at least 4 hours per week	27 (38.6)	48 (59.3)	
Active sports activities/heavy work	30 (42.9)	13 (16.0)	
Competitive sports	2 (2.9)	0	

Table S1. Participant characteristics for healthy control and osteoarthritis (OA) groups. Continuous variables† are presented as mean ± standard deviation (SD), and categorical variables‡ as number (percentage). P values reflect comparisons between groups using independent t-tests for continuous variables and chi-square tests for categorical variables. The OA group was significantly older and had higher body mass index (BMI) than controls, while sex distribution and smoking status did not differ between groups. Differences were also observed in alcohol consumption, education level, work status, and physical activity. Participant characteristics are derived in part from previously published cohort data (25).

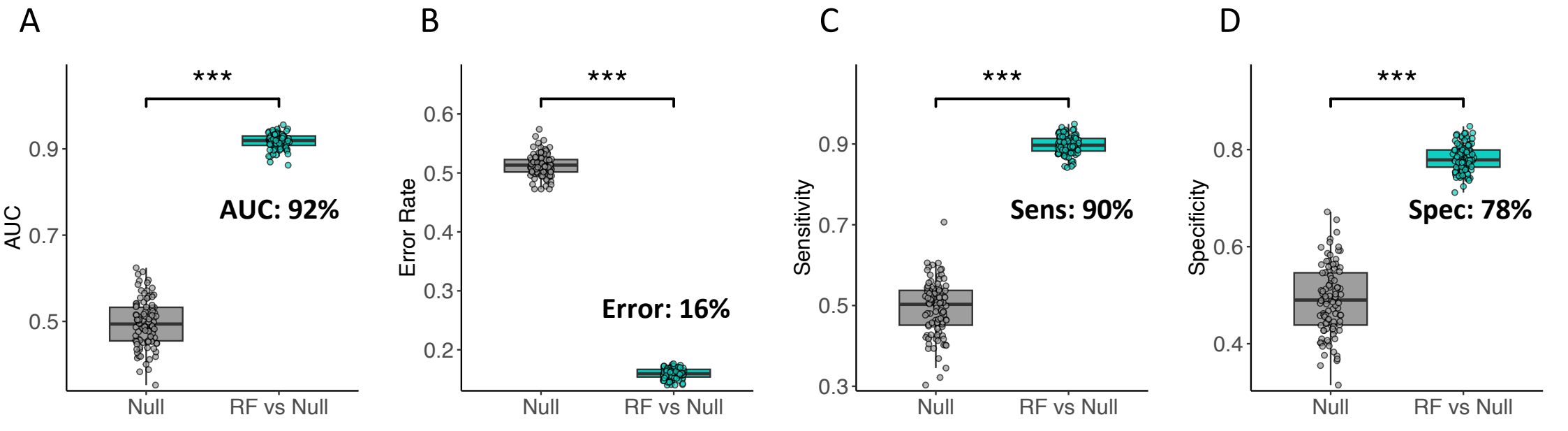

Figure S1. Random forest validation of OA vs control classification. (A) Area under the ROC curve (AUC) for the random forest (RF) model built on the same serum–CSF–protein dataset as Figure 1, compared with a permuted null model. The RF achieved an AUC of 0.92, significantly higher than the null distribution (***p<0.001). (B) Error rate of the RF model was 16%, lower than the null model (***p<0.001). (C) Sensitivity of the RF model was 90%, again exceeding the null model (***p<0.001). (D) Specificity of the RF model was 78%, higher than the null model (***p<0.001). These results show that an orthogonal machine learning approach reproduces the discrimination seen with OPLS-DA, supporting the robustness of the biofluid signature.

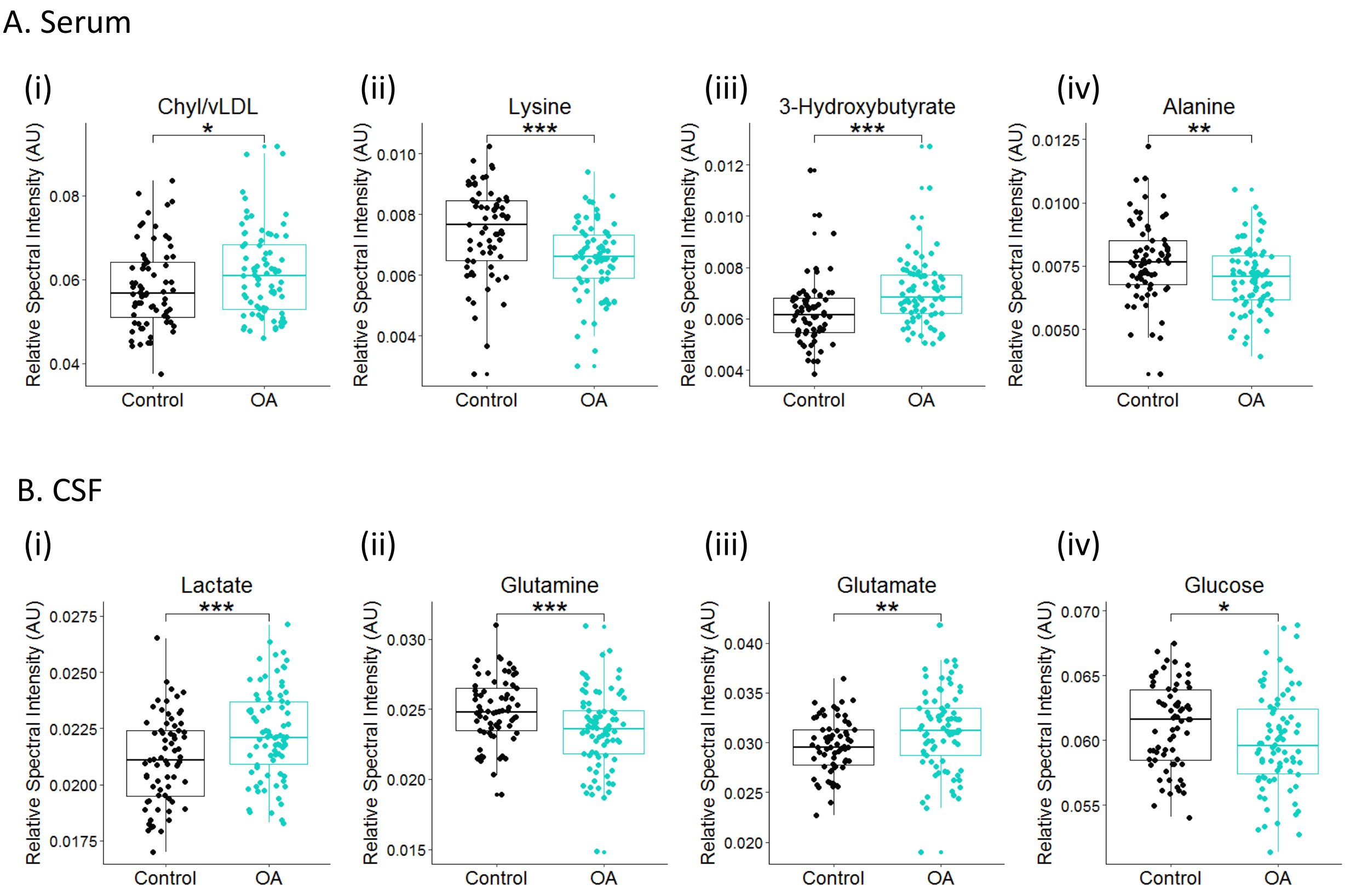

Figure S2. Box plots of top VIP metabolites from the single biofluid OPLS-DA models. (A) Serum. (i–iv) Concentrations of the highest ranking serum metabolites from the OPLS-DA model comparing OA (turquoise) with pain free controls (black): chylomicron/VLDL (i), lysine (ii), 3-hydroxybutyrate (iii) and alanine (iv). All four metabolites contributed strongly to OA–control discrimination in Figure 1A(ii) and remained significantly different on univariate testing. (B) CSF. (i–iv) Corresponding box plots for the top CSF metabolites from the CSF-only model in Figure 1B(ii): lactate (i), glutamine (ii), glutamate (iii) and glucose (iv). CSF lactate and glutamate were higher in OA, while CSF glutamine and glucose were lower. Data are shown as relative spectral intensity (arbitrary units). Asterisks denote significance: *p<0.05, **p<0.01, ***p<0.001 (unpaired t tests).

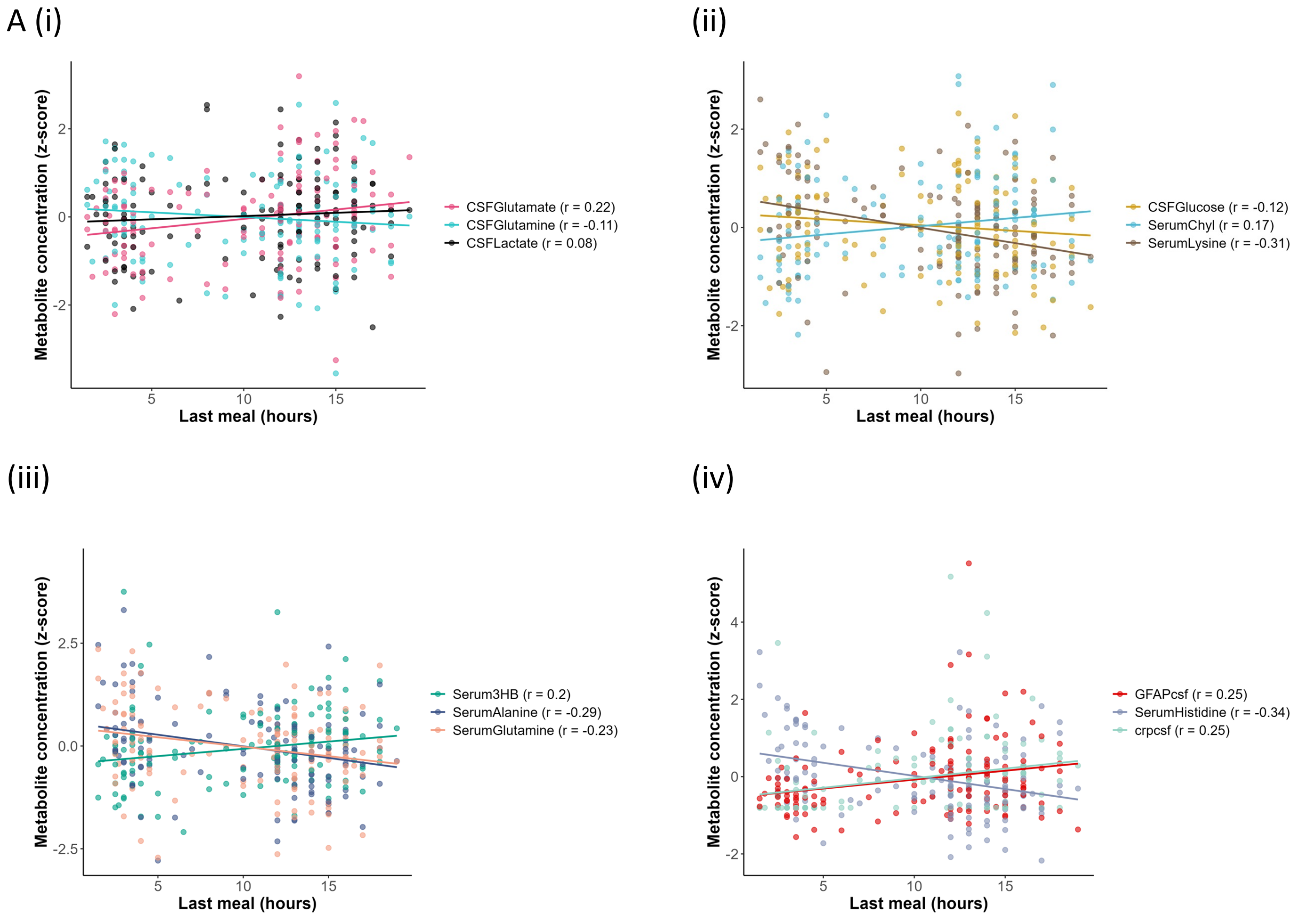

Figure S3. Associations between key OA vs healthy control discriminating metabolites and time since last meal. Scatter plots showing z-scaled metabolite concentrations plotted against time since last meal (hours) for selected serum and CSF metabolites and inflammatory markers identified as VIPs in the OPLS-DA model. Solid lines represent linear regression fits, with Pearson correlation coefficients (r) indicated. Across all panels, correlations were weak, indicating that variation in fasting duration is unlikely to be a major driver of the observed metabolic signatures.

A

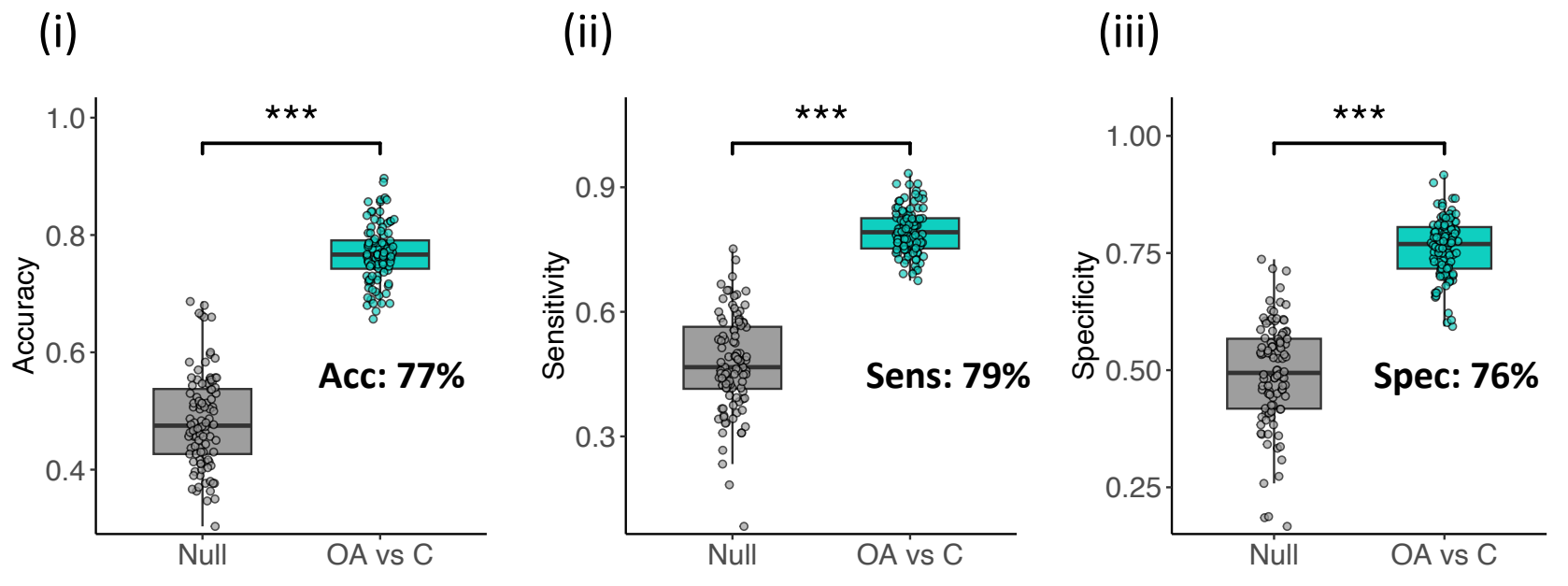

B

**VIPs all (A)**	**VIP score (all)**	**VIPs Matched cohort (B)**	**VIP score (matched)**
**Serum histidine**	2.61	**Serum histidine**	2.56
**Serum albumin (lysyl moiety)**	2.51	**Serum gp130**	2.28
**CSF CRP**	2.45	**Serum albumin (lysyl moiety)**	2.28
CSF GFAP	2.35	**CSF glucose**	2.25
Plasma IL-6	2.15	**Serum glutamine**	2.20
**CSF glucose**	2.12	Plasma TNFβ	2.05
**Serum glutamine**	2.05	**Serum citrate**	1.93
**Serum lysine**	1.99	CSF glutamine	1.81
Serum GFAP	1.88	**CSF acetate**	1.77
**CSF acetate**	1.85	Serum Lactate	1.77
CSF creatinine	1.83	CSF IL-8	1.74
CSF glutamate	1.78	**Serum lysine**	1.66
CSF TNFRII	1.70		
CSF supar	1.68		
CSF NfL	1.68		
CSF lactate	1.67		
Serum NfL	1.62		
Plasma CRP	1.53		
Serum 3-hydroxybutyrate	1.52		
**Serum citrate**	1.51		

Figure S4. Age- and BMI-matched model metrics and comparison of VIP rankings. (A) Performance of the OPLS-DA model built on the age- and BMI-matched subset of osteoarthritis (OA) and control participants. Compared with the permuted null model, the matched model retained good discrimination with (i) accuracy 77%, (ii) sensitivity 79% and (iii) specificity 76% (all ***$p<0.001$), confirming that group separation is not driven solely by demographic differences. (B) Side-by-side list of variables with the highest variable importance in projection (VIP) scores in the full, unmatched model (left) and in the matched cohort model (centre), together with VIP scores for the matched model (right). Variables that appear in both lists, such as serum histidine, serum albumin (lysyl moiety), CSF glucose, serum glutamine, serum citrate, CSF acetate and serum lysine, represent metabolites and proteins that discriminate OA from controls independently of age and BMI.

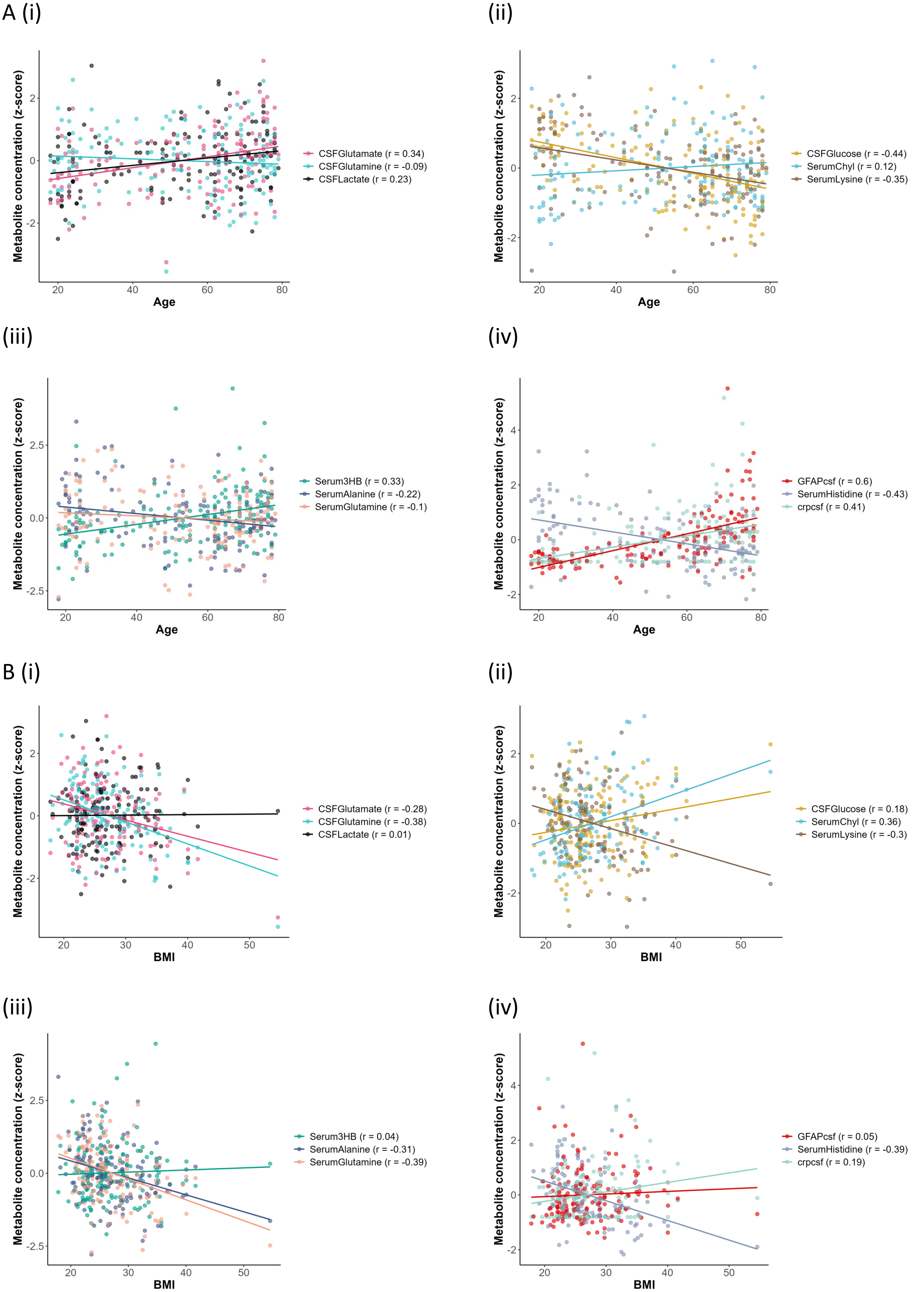

Figure S5. Associations between key OA vs healthy control discriminating metabolites and demographic variables. Scatter plots showing correlations between metabolite concentrations (scaled) and age (A) or body mass index (BMI) (B) for variables identified as top VIPs in the OPLS-DA models in Figure 1. Panels (i–iv) display VIPs, separated randomly into three per graph for visual clarity. Solid lines represent linear regression fits, with Pearson correlation coefficients (r) indicated for each variable. Overall, associations with age and BMI were modest, suggesting that the principal metabolic signatures are not primarily driven by these demographic factors.

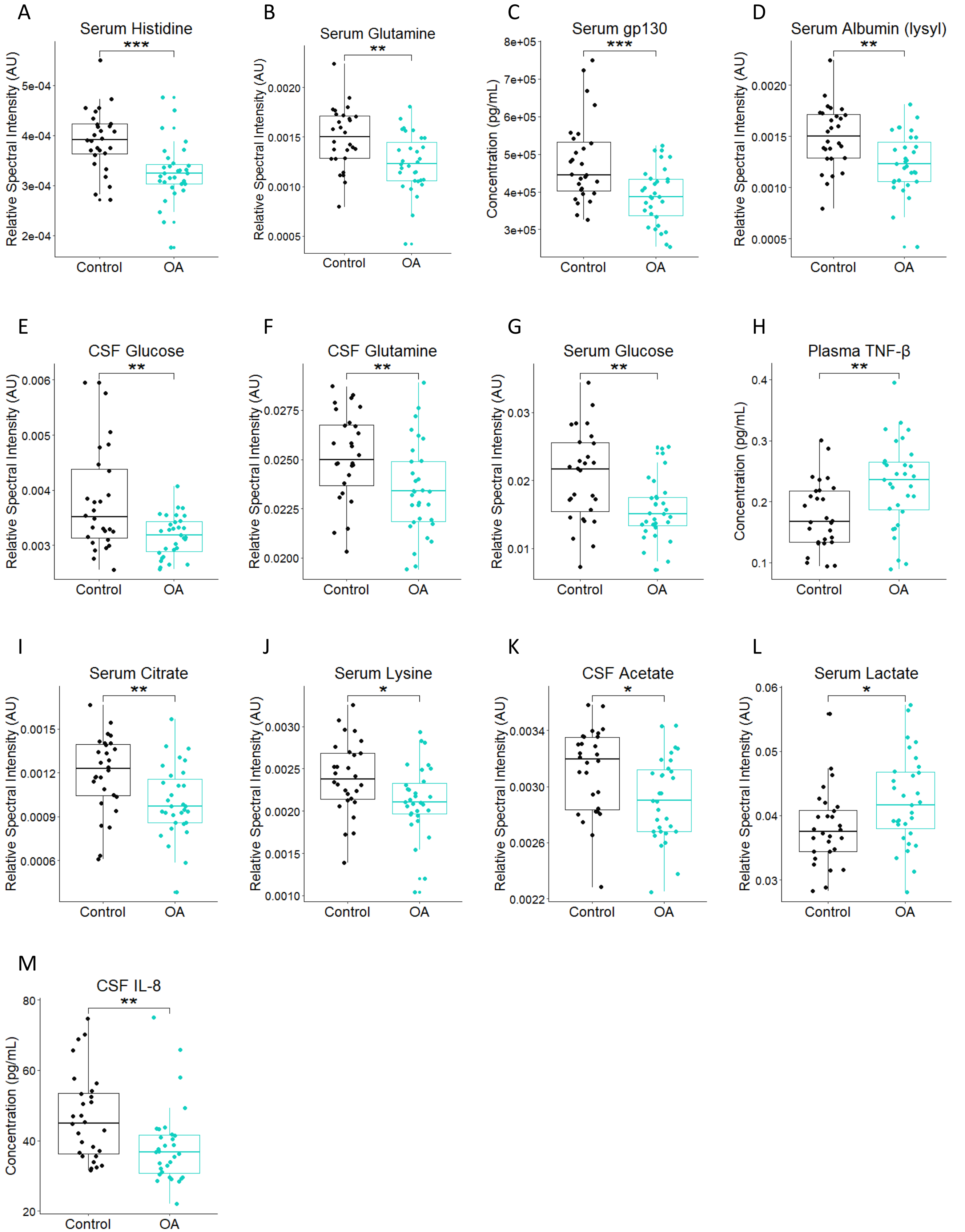

Figure S6. Key serum and CSF metabolites differentiating osteoarthritis and control participants in age- and BMI-matched subgroups. Boxplots display metabolite concentrations in osteoarthritis patients (n=31) and pain-free controls (n=28) matched for age and BMI. Metabolites were selected based on their discriminative value in multivariate analyses from the full cohorts. Asterisks indicate statistical significance based on unpaired t-tests: $*p < 0.05$, $**p < 0.01$, $***p < 0.001$, $****p < 0.0001$. These findings highlight the robustness of histidine, glutamine, and related metabolite alterations even after accounting for demographic confounders.

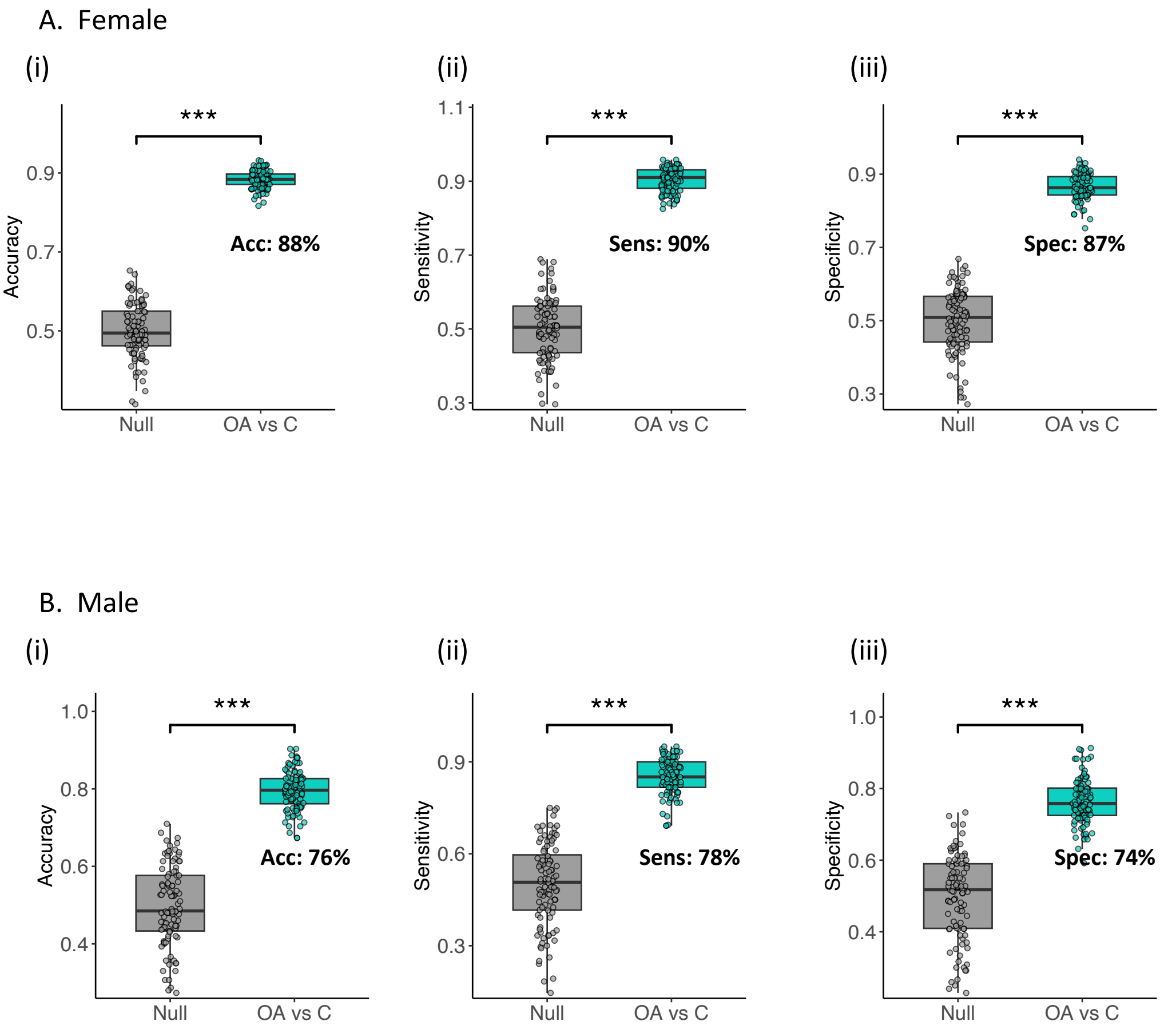

Figure S7. Sex stratified OPLS-DA model performance. (A) Females. Compared with the permuted null model, the female-only OPLS-DA model discriminated osteoarthritis (OA) from controls with (i) accuracy 88%, (ii) sensitivity 90% and (iii) specificity 87% (all ***$p<0.001$), indicating a strong metabolic–inflammatory signal in women. (B) Males. The male-only model also separated OA from controls, but with lower performance, giving (i) accuracy 76%, (ii) sensitivity 78% and (iii) specificity 74% (all ***$p<0.001$). These data support the main-figure finding that the OA biofluid signature is more pronounced in females than in males in this cohort.

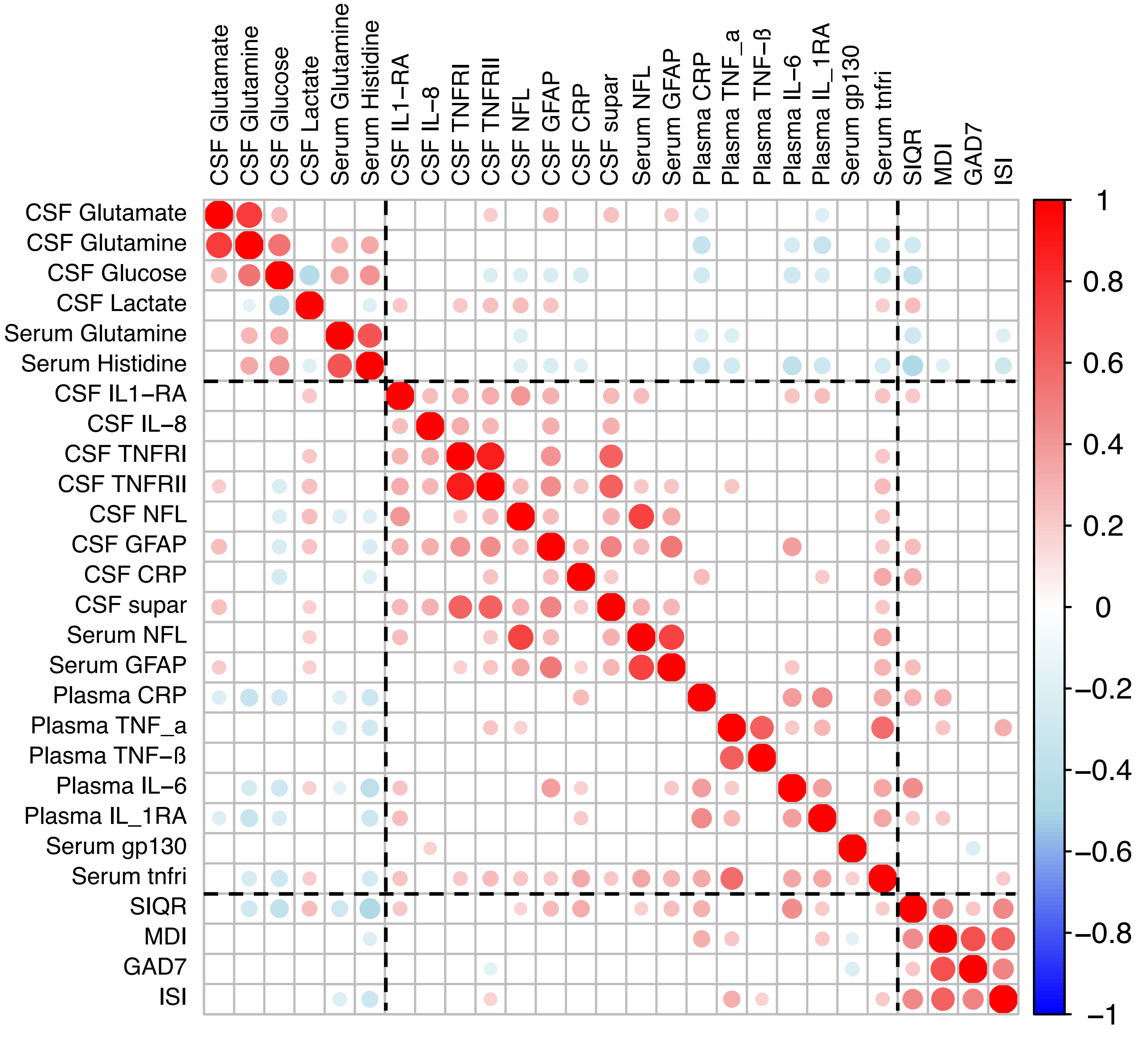

Figure S8. Correlations between key metabolites and protein biomarkers in osteoarthritis and control participants. Heatmap displaying Pearson correlation coefficients between select cerebrospinal fluid (CSF) and serum metabolites and inflammatory or neurodegenerative protein biomarkers across all study participants (OA and controls). Metabolites were selected based on their variable importance in projection (VIP) scores from the OPLS-DA model. Strong correlations were observed between glutamate, glutamine, and lactate with several central and peripheral immune markers, including TNFRI, TNFRII, IL-1RA, and GFAP, suggesting metabolic-immunological interactions relevant to OA pain pathophysiology.

Variable	Effect	Df	Sum Sq	Mean Sq	F value	p value
Age	Diagnosis	1	33080.1114	33080.11142	203.38	**<0.001**
	Sex	1	549.7755	549.7755	3.38	0.068
	Diagnosis:Sex	1	26.6939	26.6939	0.164	0.686
	Residuals	145	23584.5467	162.65205		
BMI	Diagnosis	1	198.60653	198.60653	8.763	**0.004**
	Sex	1	2.62337	2.62337	0.116	0.734
	Diagnosis:Sex	1	0.12711	0.12711	0.006	0.94
	Residuals	143	3240.90338	22.66366		
SIQR	Diagnosis	1	24792.4501	24792.45005	164.705	**<0.001**
	Sex	1	1366.58583	1366.58583	9.079	**0.003**
	Diagnosis:Sex	1	1361.10329	1361.10329	9.042	**0.003**
	Residuals	140	21073.7497	150.52678		
MDI	Diagnosis	1	284.67421	284.67421	9.239	**0.003**
	Sex	1	40.52171	40.52171	1.315	0.253
	Diagnosis:Sex	1	97.88644	97.88644	3.177	0.077
	Residuals	140	4313.91068	30.81365		
GAD7	Diagnosis	1	1.85585	1.85585	0.252	0.617
	Sex	1	8.3198	8.3198	1.128	0.29
	Diagnosis:Sex	1	10.92888	10.92888	1.482	0.226
	Residuals	140	1032.45103	7.37465		
ISI	Diagnosis	1	649.43103	649.43103	27.98	**<0.001**
	Sex	1	11.97588	11.97588	0.516	0.474
	Diagnosis:Sex	1	60.28657	60.28657	2.597	0.109
	Residuals	140	3249.46624	23.21047		
Serum Albumin	Diagnosis	1	1.52E-05	1.52E-05	32.758	**<0.001**
	Sex	1	2.20E-06	2.20E-06	4.726	**0.031**
	Diagnosis:Sex	1	2.50E-07	2.50E-07	0.541	0.463
	Residuals	144	6.70E-05	4.70E-07		
Serum Glutamine	Diagnosis	1	1.40E-06	1.40E-06	13.959	**<0.001**
	Sex	1	1.20E-07	1.20E-07	1.154	0.285
	Diagnosis:Sex	1	4.60E-07	4.60E-07	4.573	**0.034**
	Residuals	144	1.45E-05	1.00E-07		
Serum Histidine	Diagnosis	1	2.70E-07	2.70E-07	35.529	**<0.001**
	Sex	1	1.00E-08	1.00E-08	0.933	0.336
	Diagnosis:Sex	1	1.00E-08	1.00E-08	1.438	0.232
	Residuals	144	1.08E-06	1.00E-08		
CSF Glutamine	Diagnosis	1	4.48E-05	4.48E-05	6.521	**0.012**
	Sex	1	2.40E-07	2.40E-07	0.035	0.853
	Diagnosis:Sex	1	1.23E-06	1.23E-06	0.179	0.673
	Residuals	145	9.96E-04	6.87E-06		
CSF Glutamate	Diagnosis	1	1.50E-04	1.50E-04	12.776	**<0.001**
	Sex	1	2.00E-05	2.00E-05	1.4	0.239
	Diagnosis:Sex	1	3.00E-05	3.00E-05	2.972	0.087
	Residuals	145	0.00169	1.00E-05		
CSF CRP	Diagnosis	1	0.00812	0.00812	26.473	**<0.001**
	Sex	1	9.00E-05	9.00E-05	0.281	0.597
	Diagnosis:Sex	1	6.00E-05	6.00E-05	0.184	0.669
	Residuals	145	0.04447	3.10E-04		
Plasma CRP	Diagnosis	1	44.68392	44.68392	8.755	**0.004**
	Sex	1	25.22515	25.22515	4.942	**0.028**
	Diagnosis:Sex	1	4.06943	4.06943	0.797	0.373
	Residuals	145	740.07455	5.10396		

Table S2. Two-way ANOVA of clinical, metabolic and inflammatory variables by diagnosis and sex. Summary table showing the effects of diagnosis (OA vs control), sex, and their interaction (diagnosis × sex) on demographic measures (age, BMI), symptom scores (SIQR, MDI, GAD7, ISI), key serum metabolites (albumin, glutamine, histidine), CSF metabolites (glutamine, glutamate), and inflammatory markers (CSF CRP, plasma CRP). A strong main effect of diagnosis was observed for SIQR, ISI, serum histidine, CSF glutamine, CSF glutamate and CSF CRP (all $p<0.01$), confirming that these variables differ between OA and controls. Sex effects were evident for SIQR and plasma CRP, and significant diagnosis × sex interactions were detected for SIQR and serum glutamine, consistent with the sex specific patterns shown in Figure 5. P values <0.05 are shown in bold.

Variable_1	Variable_2	Pearson_r	P_raw	P_FDR
Serum Histidine	Serum Albumin	0.82312612	1.05E-37	8.03E-36
Serum Histidine	Serum Glutamine	0.59739363	1.10E-15	1.21E-14
Serum Albumin	Serum Glutamine	0.74039463	5.75E-27	1.47E-25
Serum Histidine	Serum Lysine	0.73662978	1.41E-26	3.08E-25
Serum Albumin	Serum Lysine	0.81602075	1.41E-36	7.20E-35
Serum Glutamine	Serum Lysine	0.70928322	6.22E-24	1.06E-22
Serum Histidine	Serum Citrate	0.66507006	3.01E-20	4.19E-19
Serum Albumin	Serum Citrate	0.78821398	1.37E-32	5.25E-31
Serum Glutamine	Serum Citrate	0.86204492	6.20E-45	9.49E-43
Serum Lysine	Serum Citrate	0.7238776	2.64E-25	5.05E-24
Serum Histidine	Serum gp130	0.09282017	0.26183871	0.35141512
Serum Albumin	Serum gp130	0.0050755	0.95118162	0.96378005
Serum Glutamine	Serum gp130	0.01271522	0.87809711	0.91393781
Serum Lysine	Serum gp130	-0.0211268	0.7988253	0.85180702
Serum Citrate	Serum gp130	0.02584288	0.7552088	0.82533533
Serum Histidine	CSF Acetate	0.26618222	0.00107551	0.00382683
Serum Albumin	CSF Acetate	0.28268625	0.00049927	0.00206455
Serum Glutamine	CSF Acetate	0.14054802	0.08841644	0.16700883
Serum Lysine	CSF Acetate	0.29491673	0.00027405	0.001198
Serum Citrate	CSF Acetate	0.11915191	0.14918994	0.2387329
Serum gp130	CSF Acetate	-0.0630484	0.44649513	0.54813784
Serum Histidine	CSF CRP	-0.1947502	0.01769654	0.04230579
Serum Albumin	CSF CRP	-0.2091653	0.01073172	0.0278297
Serum Glutamine	CSF CRP	-0.1413798	0.08652948	0.16700883
Serum Lysine	CSF CRP	-0.0941076	0.2552506	0.34869055
Serum Citrate	CSF CRP	-0.1017047	0.21870005	0.31567083
Serum gp130	CSF CRP	-0.0881029	0.28696523	0.38178852
CSF Acetate	CSF CRP	-0.1091132	0.18679855	0.28019783
Serum Histidine	CSF Glutamate	0.05492999	0.50873774	0.59874519
Serum Albumin	CSF Glutamate	0.08108088	0.32893084	0.4318279
Serum Glutamine	CSF Glutamate	-0.003674	0.96477294	0.97112013
Serum Lysine	CSF Glutamate	0.12903214	0.11932932	0.21229518
Serum Citrate	CSF Glutamate	0.07009832	0.39884829	0.50019499
Serum gp130	CSF Glutamate	-0.0485516	0.55923665	0.64333239
CSF Acetate	CSF Glutamate	0.39407546	7.87E-07	5.74E-06
CSF CRP	CSF Glutamate	0.15798317	0.05599022	0.11576357
Serum Histidine	CSF Glutamine	0.33305848	3.76E-05	0.00019819
Serum Albumin	CSF Glutamine	0.36331536	6.10E-06	3.89E-05
Serum Glutamine	CSF Glutamine	0.22293369	0.00664567	0.01898834
Serum Lysine	CSF Glutamine	0.33915074	2.64E-05	0.0001445
Serum Citrate	CSF Glutamine	0.2271022	0.00567135	0.01701405
Serum gp130	CSF Glutamine	-0.0437737	0.59857588	0.678386
CSF Acetate	CSF Glutamine	0.61627525	9.66E-17	1.23E-15
CSF CRP	CSF Glutamine	-0.1225538	0.13919865	0.22939772
CSF Glutamate	CSF Glutamine	0.76443079	1.96E-29	6.00E-28
Serum Histidine	SIQR	-0.4777013	1.84E-09	1.65E-08
Serum Albumin	SIQR	-0.426101	1.24E-07	9.51E-07
Serum Glutamine	SIQR	-0.3084712	0.00018781	0.00092694
Serum Lysine	SIQR	-0.3054119	0.00021907	0.00104744
Serum Citrate	SIQR	-0.2667851	0.00133019	0.00462542
Serum gp130	SIQR	-0.1296645	0.12405505	0.21816578
CSF Acetate	SIQR	-0.2609882	0.00170628	0.00555449
CSF CRP	SIQR	0.32477982	8.03E-05	0.00040964
CSF Glutamate	SIQR	0.1230879	0.14446804	0.2351448
CSF Glutamine	SIQR	-0.2857186	0.00056732	0.0022842
Serum Histidine	MDI	-0.2006481	0.01665362	0.0404445
Serum Albumin	MDI	-0.1715246	0.04124706	0.08888451
Serum Glutamine	MDI	-0.1490216	0.07672732	0.15446421
Serum Lysine	MDI	-0.0983011	0.24448056	0.34354983
Serum Citrate	MDI	-0.1346907	0.11001412	0.19833166
Serum gp130	MDI	-0.1753941	0.03681575	0.0804687
CSF Acetate	MDI	-0.1021674	0.22632756	0.32362727
CSF CRP	MDI	0.12009512	0.15456016	0.24130311
CSF Glutamate	MDI	-0.063695	0.45140763	0.54813784
CSF Glutamine	MDI	-0.1438162	0.08772746	0.16700883
SIQR	MDI	0.45271815	1.54E-08	1.24E-07
Serum Histidine	GAD7	0.01549033	0.85482308	0.89580774
Serum Albumin	GAD7	-0.0073211	0.93109179	0.95608754
Serum Glutamine	GAD7	0.04754903	0.57416922	0.65558127
Serum Lysine	GAD7	0.03914433	0.64371349	0.71889171
Serum Citrate	GAD7	0.02497355	0.76798637	0.83334691
Serum gp130	GAD7	-0.2212964	0.00812958	0.02261501
CSF Acetate	GAD7	-0.0352601	0.67697976	0.75056451
CSF CRP	GAD7	-0.0020327	0.98084567	0.98084567
CSF Glutamate	GAD7	0.05509674	0.51488812	0.60135788
CSF Glutamine	GAD7	0.0114103	0.89279215	0.92295405
SIQR	GAD7	0.20914367	0.01249333	0.03135893
MDI	GAD7	0.68790318	3.18E-21	4.86E-20
Serum Histidine	ISI	-0.3011427	0.00027082	0.001198
Serum Albumin	ISI	-0.2911342	0.00043966	0.00186854
Serum Glutamine	ISI	-0.2091222	0.01250258	0.03135893
Serum Lysine	ISI	-0.2393621	0.00411844	0.01285963
Serum Citrate	ISI	-0.1894178	0.02396418	0.05472418
Serum gp130	ISI	-0.109014	0.19655713	0.29197322
CSF Acetate	ISI	-0.0392081	0.64317301	0.71889171
CSF CRP	ISI	0.09682848	0.25165371	0.34687403
CSF Glutamate	ISI	0.12646039	0.13370317	0.22479763
CSF Glutamine	ISI	-0.0640102	0.449167	0.54813784
SIQR	ISI	0.4675287	4.46E-09	3.79E-08
MDI	ISI	0.61836155	2.42E-16	2.84E-15
GAD7	ISI	0.49627998	3.37E-10	3.22E-09
Serum Histidine	WPI	-0.3578236	8.01E-06	4.90E-05
Serum Albumin	WPI	-0.3400794	2.35E-05	0.00013307
Serum Glutamine	WPI	-0.2970214	0.0002465	0.00114287
Serum Lysine	WPI	-0.2711738	0.00085702	0.00319815
Serum Citrate	WPI	-0.2599631	0.00141871	0.00482363
Serum gp130	WPI	-0.1264061	0.12579282	0.21870797
CSF Acetate	WPI	-0.2278266	0.00535524	0.01638703
CSF CRP	WPI	0.26817291	0.0009829	0.00358055
CSF Glutamate	WPI	0.22271066	0.00670177	0.01898834
CSF Glutamine	WPI	-0.1143329	0.16792407	0.25692382
SIQR	WPI	0.51746378	4.28E-11	4.37E-10
MDI	WPI	0.21861433	0.0089552	0.02446688
GAD7	WPI	0.11852064	0.16007782	0.247393
ISI	WPI	0.35965509	1.10E-05	6.49E-05
Serum Histidine	Total NRS	-0.2309179	0.06009847	0.12260089
Serum Albumin	Total NRS	-0.2839272	0.01988824	0.04610455
Serum Glutamine	Total NRS	-0.3101426	0.01064409	0.0278297
Serum Lysine	Total NRS	-0.1425508	0.24983183	0.34687403
Serum Citrate	Total NRS	-0.2150255	0.08056096	0.16007567
Serum gp130	Total NRS	-0.0303681	0.80726809	0.85180702
CSF Acetate	Total NRS	0.1190489	0.3372883	0.43733144
CSF CRP	Total NRS	-0.2132861	0.08310121	0.16300622
CSF Glutamate	Total NRS	0.14519309	0.24475119	0.34354983
CSF Glutamine	Total NRS	0.08838779	0.48035667	0.57086103
SIQR	Total NRS	0.5345724	3.77E-06	2.62E-05
MDI	Total NRS	0.2864451	0.01971548	0.04610455
GAD7	Total NRS	0.18387359	0.13943783	0.22939772
ISI	Total NRS	0.26323387	0.03272179	0.07362403
WPI	Total NRS	-0.1528432	0.2169042	0.31567083
Serum Histidine	Oxford Score	0.19201832	0.09214463	0.1719284
Serum Albumin	Oxford Score	0.27786796	0.01377192	0.03398555
Serum Glutamine	Oxford Score	0.19096458	0.09397842	0.17323733
Serum Lysine	Oxford Score	0.18229014	0.11018426	0.19833166
Serum Citrate	Oxford Score	0.16312851	0.15357122	0.24130311
Serum gp130	Oxford Score	-0.0809093	0.4813142	0.57086103
CSF Acetate	Oxford Score	0.09002155	0.43315399	0.5388013
CSF CRP	Oxford Score	-0.1713408	0.13363375	0.22479763
CSF Glutamate	Oxford Score	0.35649244	0.00146115	0.0048599
CSF Glutamine	Oxford Score	0.37960396	0.00066182	0.00259636
SIQR	Oxford Score	-0.5082315	4.43E-06	2.95E-05
MDI	Oxford Score	-0.2321104	0.04815481	0.1009272
GAD7	Oxford Score	0.0295438	0.80404179	0.85180702
ISI	Oxford Score	-0.1010309	0.39505367	0.49953067
WPI	Oxford Score	0.04711098	0.68211191	0.75081383
Total NRS	Oxford Score	-0.3179563	0.00927826	0.02490481
Serum Histidine	EQ VAS	0.22512641	0.04751508	0.1009272
Serum Albumin	EQ VAS	0.15304776	0.18097648	0.27415249
Serum Glutamine	EQ VAS	0.16462052	0.14979319	0.2387329
Serum Lysine	EQ VAS	0.02899707	0.80103067	0.85180702
Serum Citrate	EQ VAS	0.10140165	0.37703302	0.4807171
Serum gp130	EQ VAS	0.12860361	0.26180893	0.35141512
CSF Acetate	EQ VAS	-0.0816333	0.47738634	0.57086103
CSF CRP	EQ VAS	-0.142436	0.21349784	0.31408817
CSF Glutamate	EQ VAS	0.00833225	0.94266517	0.96151848
CSF Glutamine	EQ VAS	0.07362645	0.52453747	0.60798661
SIQR	EQ VAS	-0.3865191	0.00073091	0.00279571
MDI	EQ VAS	-0.2469539	0.03517834	0.07800414
GAD7	EQ VAS	-0.1092551	0.35750849	0.45965377
ISI	EQ VAS	-0.1155715	0.33022134	0.4318279
WPI	EQ VAS	-0.171874	0.13241046	0.22479763
Total NRS	EQ VAS	-0.3316127	0.00652843	0.01898834
Oxford Score	EQ VAS	0.33776499	0.00249199	0.00794322

Table S3. Pairwise correlations between key metabolites, inflammatory markers, and clinical measures. Pearson correlation coefficients (r) are shown for selected serum and CSF metabolites, protein biomarkers, and clinical variables. For each pair, unadjusted (P_raw) and FDR-adjusted (P_FDR) P values are provided (Benjamini–Hochberg correction). Strong correlations among several serum metabolites (e.g. histidine, albumin, glutamine, lysine, citrate) suggest shared metabolic pathways rather than independent signals, whereas weaker associations with inflammatory markers and some CSF variables indicate that not all features are collinear.
